# Hepatoprotective and pancreatoprotective effects of *Arctium lappa* root extract in valproic acid–induced toxicity: biochemical, histopathological, and immunohistochemical analysis

**DOI:** 10.3389/fphar.2026.1883451

**Published:** 2026-10-16

**Authors:** Mukul Shyam, Sabina Evan Prince

**Affiliations:** Department of Biotechnology, School of Biosciences and Technology, VIT University, Vellore, Tamil Nadu, India

**Keywords:** Burdock root, hepatotoxicity, pancreatitis, phytochemical, valproic acid

## Abstract

**Introduction:**

Valproic acid (VPA) is a widely used antiepileptic drug; however, its clinical use is limited by hepatotoxicity and pancreatitis, primarily mediated through oxidative stress and inflammation. *Arctium lappa* root extract (ALRE) has been reported to possess antioxidant, anti-inflammatory, and cytoprotective properties that may confer protection against VPA-induced organ toxicity.

**Methods:**

Phytochemical profiling of ALRE was performed using GC-MS and HPLC. *In vitro* cytotoxic effects were assessed in Hep3B and PANC-1 cancer cell lines, while safety was assessed in THLE-2 normal hepatic epithelial cells and H6C7 normal pancreatic ductal epithelial cells using the MTT assay. The hepatoprotective and pancreatoprotective effects were investigated in VPA-induced toxicity in male Wistar albino rats through biochemical, oxidative stress, and histopathological analyses.

**Results:**

ALRE exhibited low cytotoxicity towards THLE-2 and H6C7 cells, with IC50 values of 1,084.92 and 1,174.62 μg/mL, respectively. VPA administration markedly increased serum levels of ALT (127.67 ± 4.34 U/L), AST (153.67 ± 4.91 U/L), ALP (178.67 ± 5.21 U/L), GGT (7.67 ± 0.09 U/L), amylase (47.33 ± 2.91 U/L), and lipase (49.00 ± 3.79 U/L) compared with the control group (p < 0.05). These enzyme levels were considerably lowered by ALRE administration, especially at 500 mg/kg. Furthermore, ALRE restored antioxidant defences (SOD, CAT, and GSH), malondialdehyde levels were decreased, and the histological architecture of the liver and pancreas was improved.

**Conclusion:**

ALRE effectively attenuated VPA-induced hepatic and pancreatic injury through antioxidant and cytoprotective mechanisms while exhibiting minimal toxicity towards normal hepatic and pancreatic cells. These findings support the potential therapeutic application of *A. lappa* as a natural protective against VPA-associated hepatotoxicity and pancreatitis. Further studies are warranted to elucidate the molecular mechanisms and pharmacokinetic interactions underlying the protective effects of ALRE.

## Graphical Abstract

**Figure:**
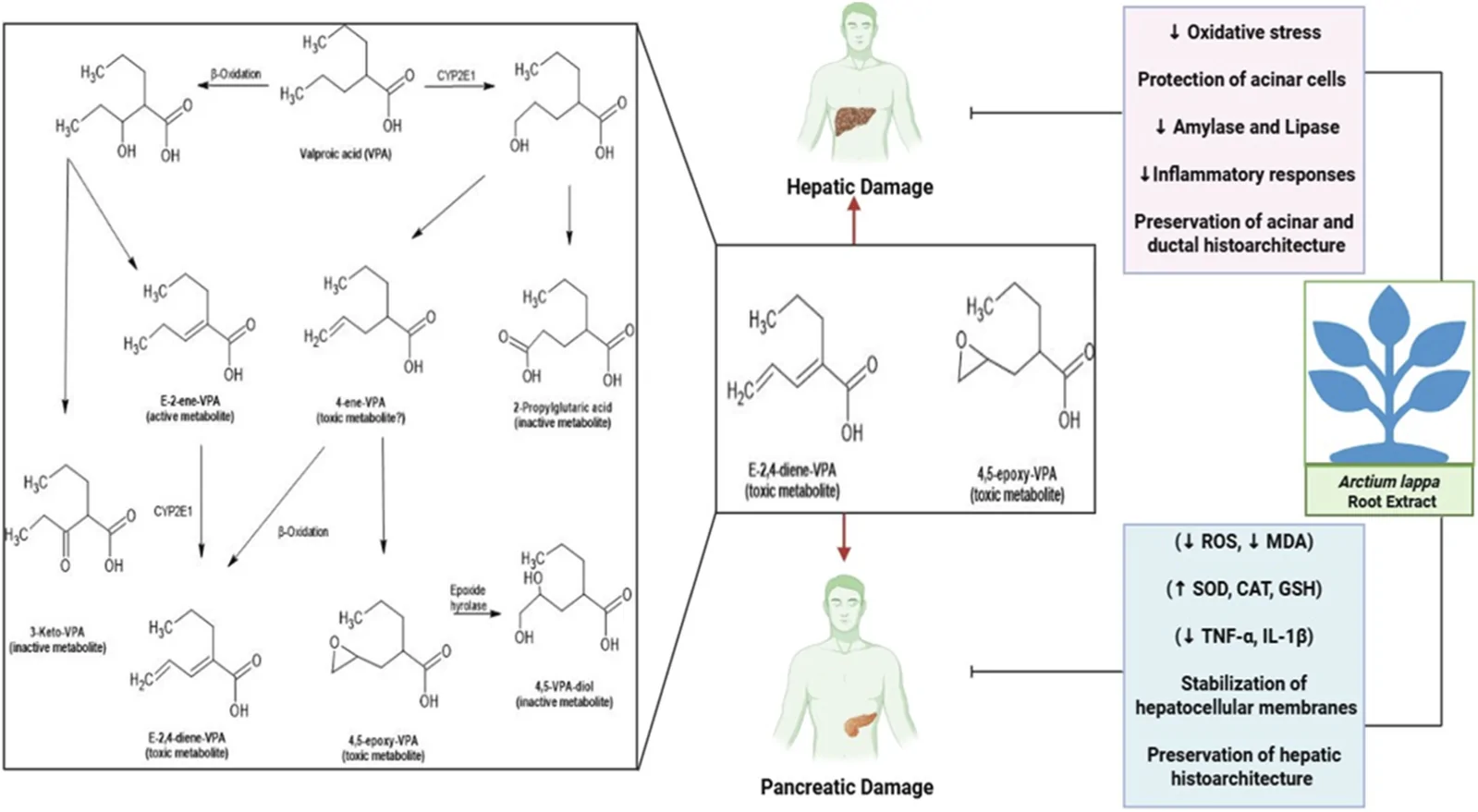

## 1 Introduction

Epilepsy is a common neurological disorder that often requires long-term treatment with antiseizure medications (Thijs et al., 2019). Valproic acid (VPA) is a broad-spectrum antiseizure medication widely used for the management of epilepsy as well as other conditions, including bipolar disorder and migraine prophylaxis (Romoli et al., 2018). Despite its clinical efficacy, VPA is associated with serious adverse effect, among which hepatotoxicity and acute pancreatitis are the most significant. VPA undergoes extensive hepatic metabolism, generating toxic metabolites such as 4-ene-VPA and 2,4-diene-VPA that contribute to mitochondrial dysfunction, oxidative stress, and inflammatory responses, ultimately leading to liver and pancreatic injury (Nanau and Neuman, 2013).

VPA, which has a broad spectrum of efficacy for the treatment of epilepsy and other FDA-approved indications such as bipolar disorder and migraine prophylaxis, is one of the most used antiepileptic drugs (AEDs). Although there are numerous benefits to prescribing VPA, it is associated with several adverse events that can be very severe, with the most significant being hepatotoxicity and acute pancreatitis, as illustrated in Supplementary Material 1, which includes the types and incidence of these adverse events, their geographic distribution, and the distributions of these events by patient age. VPA undergoes extensive metabolism in the liver and follows a biological pathway that involves the glucuronidation of the drug and its metabolites; mitochondrial beta-oxidation; and cytochrome P450-mediated oxidation (Tan et al., 2010). In addition, the accumulation of toxic metabolites (4-ene-VPA and 2,4-diene-VPA) caused by VPA metabolism disrupts mitochondrial fatty acid metabolism and leads to lipid accumulation, depletion of coenzyme A (CoA), inhibition of carnitine palmitoyl transferase I (CPT-I), and ultimately hepatocellular injury (Tang et al., 1995; Neuman et al., 2001). In addition, oxidative stress and depleting of antioxidant defences in the pancreas of patients treated with VPA result in lipid peroxidation and injury to pancreatic tissue (Talwar, 1994; Ray et al., 2015).

L-carnitine has been widely investigated as a protective agent against VPA-induced toxicity because of its ability to improve mitochondrial β-oxidation, reduce oxidative stress, and mitigate the accumulation of toxic metabolites. Experimental and clinical studies have demonstrated that L-carnitine can attenuate VPA-induced hepatic injury and metabolic disturbance (Lheureux and Hantson, 2009; Gziut and Thanacoody, 2024). Therefore, L-carnitine was included in the present study as positive control to compare the hepatopancreatoprotective efficacy of ALRE.

Antioxidant therapeutic approaches are gaining popularity because of the role of oxidative stress in causing organ damage with VPA. Researchers have focused on finding new medications to relieve the adverse effects of hepatotoxicity and pancreatitis related to VPA. There have been studies where pharmaceutical agents, including the antioxidants L-carnitine, N-acetylcysteine, and vitamin E, were tested as potential ways of improving mitochondrial function, reducing oxidative stress on the body, and preventing hepatic failure caused by VPA therapy (Said and El-Agamy, 2011; Abdella et al., 2014; Okumura et al., 2021). In addition to synthetic antioxidant products, natural products and plant extracts have also been assessed by a few studies to determine their effectiveness as protective agents against organ damage from VPA. Natural extracts such as curcumin, resveratrol, silymarin, and ellagic acid have been shown in experimental models of VPA-induced organ damage to provide protection to the liver and decrease inflammation (Abdelkader et al., 2020; Gheena et al., 2022; Terim Kapakin et al., 2025). The mechanism behind these protective benefits appears to be related to their capacity to reduce oxidative stress and improve the antioxidant capacity of the body.

Burdock (*Arctium lappa*), an Asteraceae family traditional herbal medicine, has high levels of phytochemicals such as inulin, lignans, polyphenols, and bioflavonoids. Pre-clinically, Burdock extracts have shown hepatoprotective, anti-inflammatory, and antioxidant effects in multiple models. Specifically, Burdock has been shown to improve hepatic toxicity from a variety of toxicants including cadmium, lead, chromium, and acetaminophen, through anti-inflammatory and antioxidant mechanisms. In addition, *A. lappa* may help protect against the loss of important antioxidant enzymes, including glutathione (GSH), superoxide dismutase (SOD), glutathione peroxidase (GPx), and catalase (CAT), therefore decreasing the production of reactive oxygen species (ROS). By balancing the oxidative environment, Burdock blocks the activation of the major pro-inflammatory transcription factor NF-κB and promotes the activation of the cellular antioxidant defence protein Nrf-2. Furthermore, *A. lappa* can protect against mitochondrial dysfunction, lipid peroxidation, breakdown of the mitochondrial electron transport chain, disruption of calcium homeostasis, inhibition of the NOX pathway, and activation of pro-apoptotic caspases (Shyam and Sabina, 2024; Yang et al., 2025).

Previous studies indicate that *A. lappa* has beneficial properties as both an antioxidant and hepatoprotective agent when tested against toxicities of acetaminophen, cadmium, lead, chromium (VI), and radiation. However, no research has been performed evaluating how *A. lappa* protects the liver and pancreas against damage caused by VPA. Based on the established antioxidant, anti-inflammatory, and cytoprotective properties of *A. lappa*, we hypothesized that the hydroalcoholic *A. lappa* root extract (ALRE) would attenuate VPA-induced hepatotoxicity and pancreatitis by reducing oxidative stress, suppressing inflammatory responses, and preserving the structural and functional integrity of hepatic and pancreatic tissues. Therefore, the present study aimed to evaluate the hepatoprotective and pancreatoprotective effects of ALRE in a rat model of VPA-induced toxicity using biochemical, histopathological, and immunohistochemical approaches. Accordingly, the primary outcome measures included serum liver function markers (ALT, AST, ALP, and GGT), pancreatic enzymes (amylase and lipase), oxidative stress biomarkers (SOD, CAT, GSH, and MDA), and the expression of inflammatory mediators (TNF-α and IL-1β). Histopathological and immunohistochemical analyses were performed as supportive secondary outcome measures to further characterize tissue protection.

## 2 Materials and methods

### 2.1 Materials

Valproic acid (Catalogue No: PA 22 03000; CAS No: 99-66-1; molecular formula C_8_H_16_O_2_; molecular weight 144.21) was procured from Pharmaffiliates Pvt. Ltd., India, and stored at 2 °C–8 °C until use. L-carnitine (Catalogue No: 24352; CAS No: 541-15-1; extrapure, 99% purity; molecular weight 161.2; Batch No: S392907) was obtained from SRL (Sisco Research Laboratories Pvt. Ltd., Mumbai, India). *Arctium lappa* (Burdock) powder (Batch No: AV/AL060324) was procured from Kshipra BioTech Pvt. Ltd., India. We used commercially available diagnostic kits to measure serum biochemical parameters, following the instructions from the manufacturers. We used AUTOSPAN® Liquid Gold IFCC kinetic assay kits (REF No: 715520-60) from Arkray Healthcare Pvt. Ltd. in India to measure AST and ALT. We used the TRUEchemie Alkaline Phosphatase Test Kit (SR) (IFCC method; REF No: ADX201) from TRUchemie, India, to estimate ALP. We used the TRUEchemie Alpha Amylase (CNPG3 method; REF No: AX261) and TRUEchemie Lipase (Colorimetric method; REF No: ADX306) kits to measure serum alpha-amylase and lipase, respectively. We used the TRUEchemie GGT Test Kit (IFCC kinetic method; REF No: ADX191, TRUchemie, India) to measure serum gamma-glutamyl transferase (GGT) activity to measure GGT levels. All other chemicals and reagents used were of analytical grade and procured from standard commercial suppliers.

### 2.2 Preparation of *Arctium lappa* root extract

A Soxhlet apparatus was used to help extract naturally occurring chemicals from the plant materials chosen to help get the most efficient extraction using organic solvents through the process of hot continuous extraction. The root of *A. lappa* was dried out and ground into a powder and placed in a cellulose extraction thimble that was placed in the main chamber (boiler) of the Soxhlet extractor. Different types of organic solvents were each evaluated for their solvent-specific efficiencies to extract the phytoconstituents using methanol, ethanol, acetone, distilled water, and a hydroalcoholic solvent (50% methanol and 50% water (v/v)) for their extraction abilities. Each individual solvent was added to a round-bottom flask that is connected to the Soxhlet apparatus and heated using a heating mantle. A heating mantle applies heat to the bottom of the round-bottom flask and at the same time heats up whatever is inside the round-bottom flask. Heat causes the organic solvent molecules to turn to steam (gas) and travel up to the top of the Soxhlet apparatus, cool, return to the liquid state, and drip down into the cellulose extraction thimble containing the plant material. After several minutes of heating, the round-bottom flask started to fill with a solution of the solvent and the dissolved phytoconstituents from the plant. After the solution had filled the chamber of the Soxhlet extractor to the point where it reached the overflow point, the siphon arm would siphon the contents of the filled chamber back into the round-bottom flask. The cycle of heating and filling the chamber continued for 6–8 h after which time the siphoned solvent appeared colourless. The organic solvent extracts obtained from the extraction process were concentrated under low pressure using a rotary evaporator. After extraction, the crude extracts were dried and stored in sealed amber vials at 4 °C until they were ready for phytochemistry and biological testing. The percentage yield of each extract was calculated using the formula below:

$$Percentage\;Yield\left(\%\right)=Weight\;of\;crude\left(g\right)÷Weight\;of\;Plant\;Material\left(g\right)\times 100$$

### 2.3 Phytochemical profiling

#### 2.3.1 GCMS analysis

We used a PerkinElmer Clarus 680 gas chromatograph and a PerkinElmer Clarus 600 mass spectrometer to do gas chromatography–mass spectrometry (GC–MS) analysis of the extract. An Elite-5MS capillary column (5% phenyl–95% dimethylpolysiloxane; 30 m × 0.25 mm i.d., 0.25 μm film thickness) was used to separate the chemicals. The carrier gas was helium, which flowed at a steady rate of 1.0 mL/min. A 1 μL sample was injected in split mode (10:1), and the injector was kept at 250 °C. The oven temperature program started at 60 °C and stayed there for 2 min. It then rose by 10 °C every minute until it reached 280 °C and stayed there for 10 min. The whole process took about 30 min. The mass spectrometric detection was done in electron ionization (EI) mode at 70 eV. The temperatures of the ion source and transfer line were kept at 230 °C and 280 °C, respectively. Spectra were taken in scan mode across a mass range of m/z 40–600.

By comparing their mass spectra to those in the NIST mass spectral library and matching retention durations, we were able to provisionally identify the compounds.

#### 2.3.2 HPLC analysis

A Shimadzu HPLC system with a Phenomenex C18 reversed-phase column and a UV detector was used for the high-performance liquid chromatography (HPLC) analysis. 20 μL of the material was introduced into the HPLC system after it had been dissolved in HPLC-grade methanol and filtered through a 0.22 µm membrane filter. Using a mobile phase of methanol: water (60:40, v/v) at a flow rate of 0.5 mL/min, chromatographic separation was accomplished under isocratic conditions. At 280 nm, the eluents were observed. With a few small adjustments, the chromatographic conditions were taken from the published method. LabSolutions LC software was used to collect and process the chromatographic data (Al-Shammaa et al., 2017). By comparing their retention periods with those documented in the literature under comparable chromatographic conditions, the phytochemical ingredients were tentatively identified.

### 2.4 DPPH antioxidant assay

The antioxidant activity of the extract was evaluated using the 2,2-diphenyl-1-picrylhydrazyl (DPPH) radical scavenging assay. The principle of this assay is based on the ability of antioxidants to donate a hydrogen atom or proton to the stable DPPH free radical, reducing it to a non-radical form. This reduction results in a color change from deep purple to yellow, which can be quantified by measuring the decrease in absorbance at 517 nm. A 0.2 mM DPPH solution was prepared in 80% methanol. Briefly, 4.0 mL of the sample at various concentrations was mixed with 1.0 mL of the 0.2 mM DPPH solution. The reaction mixture was shaken vigorously and incubated in the dark at room temperature for 30 min. After incubation, the absorbance was measured at 517 nm using a UV–Visible spectrophotometer. Butylated hydroxytoluene (BHT) was used as the positive control (Shimada et al., 1992). The DPPH radical scavenging activity was expressed as the percentage inhibition and calculated using the following equation:

$$\%\text{ Inhibition}=\left[\left(A_{0}-A_{1}\right)\;/\;A_{0}\right]\times 100$$

where *A*_*0*_ is the absorbance of the control and *A*_*1*_ is the absorbance of the sample.

### 2.5 ABTS^+^ radical scavenging assay

The antioxidant activity of the extract was determined using the 2,2′-azinobis-(3-ethylbenzothiazoline-6-sulfonic acid) (ABTS^+^) radical scavenging assay according to the method described by (Gião et al., 2007). The assay is based on the generation of the ABTS^+^ chromophore through the oxidation of ABTS with potassium persulfate. This method is applicable for evaluating the antioxidant activity of both hydrophilic and lipophilic compounds. The ABTS^+^ working solution was prepared by mixing equal volumes of 7 mM ABTS and 2.45 mM potassium persulfate, followed by incubation in the dark at room temperature for 12–16 h to allow radical generation. Prior to use, the solution was diluted with ethanol to obtain an absorbance of 0.70 ± 0.05 at 734 nm. For the assay, 1.0 mL of the diluted ABTS^+^ solution was mixed with 10 μL of the sample at different concentrations (5–100 μg/mL), while 10 μL of ethanol served as the control. Ascorbic acid was used as the positive control. The reaction mixture was incubated for 6 min at room temperature, and the absorbance was measured at 734 nm using a UV–Visible spectrophotometer. The ABTS^+^ radical scavenging activity was expressed as the percentage inhibition and calculated using the following equation:

$$\%\text{ Inhibition}=\left[\left(A_{0}-A_{1}\right)\;/\;A_{0}\right]\times 100$$

where *A*_*0*_ is the absorbance of the control and *A*_*1*_ is the absorbance of the sample.

### 2.6 Total phenolic content

The method of determining total phenolic content was based on the Folin-Ciocalteu reagent method (Yap et al., 2019). A series of standard solutions of gallic acid, with concentrations ranging from 20 to 100 mg/L, were prepared and compared to extract samples that had been prepared using the same concentration range. The test method for both the gallic acid standard and the extract samples used aliquots of 1 mL from each of their respective standard or sample solutions mixed with 5 mL of 10% Folin-Ciocalteu reagent. After 5 minutes, 4 mL of 7.5% sodium carbonate (Na_2_CO_3_) solution were added to the mixture, and the resulting reaction mixture was vortexed and allowed to incubate for 1 hour in a dark room at room temperature to allow the color to develop, after which the absorbance was measured at a wavelength of 765 nm using a UV-visible spectrophotometer. The absorbance data acquired from the gallic acid standards was used to generate a calibration curve. Lastly, total phenolic content (TPC) values are based upon the dry weight of the extract samples and expressed as mg gallic acid equivalents per g dry root (mgGAE/g dry root).

### 2.7 Total flavonoid content

Aluminium chloride colorimetric method was used to determine the total flavonoid content of the extract (Chang et al., 2002). The series of standard solutions of quercetin were made in quintuplicate at concentrations of 20, 40, 60, 80, and 100 mg/L. Samples of the extract were diluted to the corresponding concentrations for measurement. Samples or standards (1 mL of each) were mixed with 2% aluminium chloride solution (1 mL) prior to incubating at room temp for 60 min in darkness, thus allowing the formation of the flavonoid-aluminium chloride complex. The absorbance of the formed yellow complex was measured spectrophotometrically using a UV-visible spectrophotometer at 420 nm after the incubation period had passed. A standard calibration curve was generated for the quercetin solutions. The total flavonoid content (TFC) was calculated and expressed as mg quercetin equivalent per gram of dry root (mg QE/g dry root) based on dry weight.

### 2.8 MTT cell viability assay using Hep3B Cells and PANC-1 cells

We got the human hepatocellular carcinoma cell line (Hep3B) and the human pancreatic cancer cell line (PANC-1) from the National Centre for Cell Science (NCCS) in Pune, India. The request numbers were 506/2025–26 and 1,398/2024–25, respectively. NCCS used the Applied Biosystems 3,500 Genetic Analyzer to analyse both cell lines and the AmpFlSTR® Identifiler® Plus PCR Amplification Kit (Applied Biosystems) to authenticate them. The STR profiles matched the ATCC reference database 100% of the time. Hoechst staining and PCR-based procedures were used to make sure that both cell lines were free of *mycoplasma* contamination before they were sent out. After being revived, cells were only employed for a short time to keep their phenotypic stability and the uniformity of the experiments.

We utilized the MTT assay (3-(4,5-Dimethylthiazol-2-yl)-2,5-diphenyltetrazolium bromide) to evaluate how much the ALRE killed human Hep3B and PANC-1 cells. Hep3B was grown in Minimum Essential Medium (MEM), while PANC-1 was grown in DMEM. Both were supplemented with 10% Fetal Bovine Serum (FBS) and 1× Antibiotic-Antimycotic Solution (Himedia, India). All cultures were kept in a humidified 37 °C/5% CO_2_ incubator.

Cell lines were seeded in 96 well plates at a density of 1 × 10^4^ cells per well and then incubated for 24 h to allow attachment. After 24 h, the media was removed, and the cells gently washed with 1× PBS. The cells were then treated with various concentrations of ALRE (25, 50, 100, 250, 500, 750,1000 and 1,250 μg/mL) in serum free media and incubated for 24 h. Concentration ranges were determined based on the solubility of the crude hydroalcoholic extract and standard *in vitro* testing methods for plant extracts using MTT assays. Low concentrations (≤25/µg/mL) are used to determine baseline cell compatibility, and high concentrations to evaluate dose dependent cell death, and to determine IC50 values. Studies conducted on extracts of *A. lappa* and other phytochemicals have consistently used this concentration range; thus, the doses selected appear to be appropriate.

Although maximum cytotoxic controls such as 20% DMSO or sodium dodecyl sulfate (SDS) are commonly used employed in MTT assays, they were not included in the present study. Doxorubicin (2 μg/mL) was used as a positive control to validate assay responsiveness.

After adding treatment, the MTT solution (0.5 mg/mL in PBS) (100 µL) was added to each well. To help complete the production of formazan crystals from the active cells, the cells were placed in the incubator (37 °C) for 4 h. The MTT solution was then removed, and all wells were thoroughly rinsed with PBS. After that, DMSO (100 µL) was added to each well to dissolve any formazan crystals; and with a gentle mixing of the solution, the formazan crystals were fully dissolved. Absorbance was quantified with a microplate reader using 570 nm (used to assess cell viability based on the viability of control cells compared to treated cells) and expressed as a percentage (Florento et al., 2012). The MTT assay was conducted post a period of 24 h following treatment to determine the cytotoxic profile of the ALRE. The main aim of this research is to investigate the *in vivo* hepatoprotective and pancreatic protective activities of the extract, thus only one instance of 24-h duration was used for the preliminary *in vitro* cytotoxicity study.

### 2.9 MTT cell viability assay using normal hepatic (THLE-2) and pancreatic (H6C7) cell lines

THLE-2 (normal human liver epithelial cells) and H6C7 (normal human pancreatic duct epithelial cells), both obtained from the National Centre for Cell Science (NCCS), Pune, India, were used for additional research on the safety profile of ALRE in non-transformed cells. Both cell lines were grown at 37 °C in a humidified environment with 5% CO2 in Eagle’s Minimum Essential Medium (EMEM) supplemented with 10% FBS.

At a density of 1 × 10^6^ cells/well, cells were seeded onto 96-well plates and given a full day to adhere. The cells were then incubated for an additional 48 h after being treated with serial concentrations of ALRE (250, 500, 750, 1,000, and 1,250 μg/mL) produced in serum-free media. To verify assay responsiveness, doxorubicin (2 μg/mL) was utilized as the positive control and untreated cells as the negative control. Every therapy was carried out three times.

After the treatment time, each well received 15 µL of MTT solution (5 mg/mL), and the plates were incubated at 37 °C for 4 h to allow formazan crystals to form. The formazan crystals were then dissolved by carefully removing the MTT-containing solution and replacing it with 100 µL of DMSO. A microplate reader was used to detect absorbance at 570 nm (Florento et al., 2012).

### 2.10 Animal study design

A total of thirty-six (36) healthy male Wistar albino rats aged between 9-10 weeks and weighing between 180–220 g were utilized for this study. The rats were obtained from the animal facility attached to Vellore Institute of Technology (VIT), located at Vellore, Tamil Nadu, India. The animal study was approved by the Institutional Animal Ethics Committee (IAEC), Vellore Institute of Technology (VIT), Vellore, Tamil Nadu, India (Approval No. VIT/IAEC/17Apr/24/26). All animal experiments were conducted in accordance with the guidelines of the Committee for the Purpose of Control and Supervision of Experiments on Animals (CPCSEA), Government of India, and are reported in accordance with the ARRIVE 2.0 guidelines. The study was conducted in accordance with the local legislation and institutional requirements. The rats had a controlled environment, which consisted of a temperature of 27 °C ± 03 °C, relative humidity between 35%–50% and a light/dark cycle of 12 h each. All rats were fed the standard pelletised lab diet and allowed free access to drinking water. After a final week of acclimatization, animals were allocated to six separate randomly selected groups (see Table 1; Figure 1) each containing six animals. The required sample size was estimated using G Power software (version 3.1.9.7) based on a one-way ANOVA design with six experimental groups, an effect size (f) of 0.40, a significance level (α) of 0.05, and a statistical power (1-β) of 0.80.The analysis indicates that a larger sample size would be required to detect moderate effect sizes. However, considering ethical constraints associated with animal experimentation and principles of 3Rs (Replacement, Reduction, and Refinement), six animals per group were selected. The sample size is consistent with previous studies employing VPA-induced toxicity models and was sufficient to detect statistically significant biochemical, histopathological, and immunohistochemical differences among the experimental groups.

**TABLE 1:** Groups and treatment description.

Group	Treatment description
Group 1 (Control)	Normal saline (untreated control group)
Group 2 (VPA)	Valproic acid (300 mg/kg b.w/day, p.o.)
Group 3 (VPA + ALRE-L)	Valproic acid (300 mg/kg b.w/day, p.o.) + *Arctium lappa* root extract (300 mg/kg b.w/day, p.o.)
Group 4 (VPA + ALRE-H)	Valproic acid (300 mg/kg b.w/day, p.o.) + *Arctium lappa* root extract (500 mg/kg b.w/day, p.o.)
Group 5 (VPA + L-Car)	Valproic acid (300 mg/kg b.w/day, p.o.) + L-Carnitine (300 mg/kg b.w/day, p.o.)
Group 6 (ALRE-H)	*Arctium lappa* root extract (500 mg/kg b.w/day, p.o.)

**FIGURE 1:**
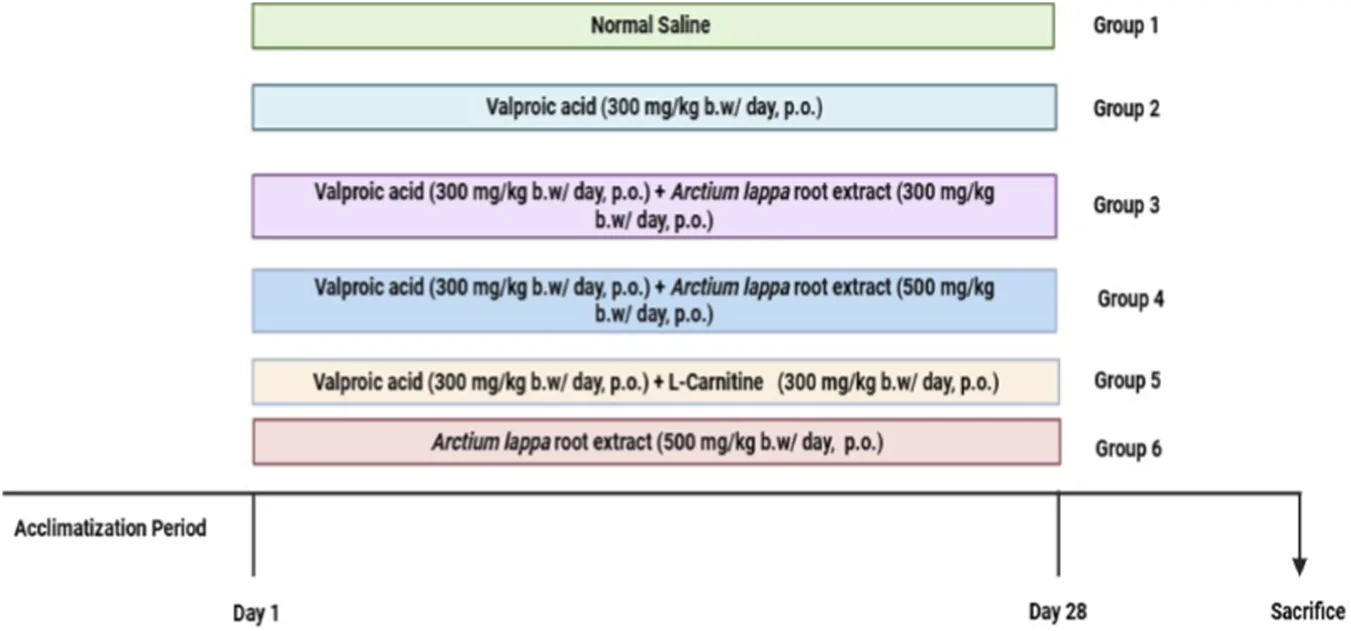
A schematic illustration of the study outlining the treatment groups, drug administration schedule, and experimental timeline for valproic acid, *Arctium lappa* root extract, and L-carnitine.

Male rats were used to minimize variability associated with hormonal fluctuations during the estrous cycle and to maintain consistency with previous studies investigating VPA-induced hepatotoxicity and pancreatitis (Becker et al., 2016).

L-carnitine was selected as the reference drug because of its established protective effects against VPA-induced toxicity through the restoration of mitochondrial-β oxidation, reduction of oxidative stress, and mitigation of toxic metabolite accumulation. Since oxidative stress and mitochondrial dysfunction are key mechanisms underlying both VPA-induced hepatotoxicity and pancreatitis, L-carnitine served as a clinically positive control in present study (Alhasaniah, 2023; Gziut and Thanacoody, 2024).

On Day 29, all animals were anesthetized using isoflurane administered via an induction chamber (3%–5%) followed by maintenance through a nose cone (1.5%–2.5%) in 100% oxygen. Upon achieving deep surgical anaesthesia, confirmed by the absence of pedal and corneal reflexes, blood was collected via cardiac puncture. Animals were then euthanized by exsanguination under anaesthesia, and the liver and pancreas were immediately dissected, rinsed in ice-cold PBS, and stored at −80 °C for biochemical, molecular, and histological analyses. The 28-day treatment period was selected based on previous reports demonstrating that repeated VPA administration for 3-4 weeks induces reproducible biochemical and histopathological manifestations of hepatotoxicity and pancreatitis in rodent models, thereby providing an appropriate time frame to evaluate the protective effects of ALRE (Abdel-Dayem et al., 2014).

The dosages of ALRE for this study of 300 mg/kg and 500 mg/kg (p.o.) were selected due to previous studies showing that *Arctium lappa* extracts were hepatoprotective; antioxidant and anti-inflammatory at these dosages. In addition, prior rodent studies of hepatotoxicity due to chemicals (acetaminophen, cadmium, chromium (VI), lead) have found dosages administered to animals ranging from 200 to 500 mg/kg to provide biochemical and histological protection from injury, with no observable signs of toxicity; this supports our choice to use these dosages in our work (de Souza Predes et al., 2014; El-Kott and Bin-Meferij, 2015; Kwon et al., 2016; Alhusaini et al., 2019). The ALRE-alone group was included at higher dose (500 mg/kg) to evaluate the safety profile of the extract at the maximum dose used in the study and to exclude any potential intrinsic effects of ALRE on hepatic and pancreatic biochemical, histological, and immunohistochemical parameters. Since the lower dose (300 mg/kg) had previously been reported to be safe in experimental models, only the higher dose was selected for the extract-alone group to minimize animal usage while ensuring assessment of safety at the highest exposure level.

In the current study, for both dosages used there were no signs of acute toxicity, such as decreased activity, abnormal grooming, behavioural abnormalities. No animals were eliminated from the study and all provided ALRE completed the entire treatment duration. Together, these findings indicate that the dosages selected are within the safe dosage range when given orally to rats.

### 2.11 Calculation of liver and pancreas index

Liver and pancreas index was calculated based on the following equation:

$$Liver\;index\;\left(\%\right)=\frac{\;Liver\;weight}{Body\;weight}\;*100$$

$$Pancreas\;index\;\left(\%\right)=\frac{\;Pancreas\;weight}{Body\;weight}\;*100$$

### 2.12 Measurement of hepatocellular enzymes

#### 2.12.1 Estimation of serum alanine aminotransferase

We used the AUTOSPAN® Liquid Gold ALT kit (IFCC kinetic method; Arkray Healthcare Pvt. Ltd., India) to estimate serum alanine aminotransferase (ALT) activity. The assay was done at 37 °C with a 1 cm light path and a main wavelength of 340 nm. In short, 1,000 µL of working ALT reagent was put into a cuvette and kept at 37 °C. Then, 100 µL of serum sample was added and mixed gently. There was a 60-s delay before the first reading, and then absorbance was measured every 30 s for three readings. We found the average change in absorbance per minute (ΔA/min) and used the kinetic factor given by the manufacturer to show ALT activity in U/L.

#### 2.12.2 Estimation of serum aspartate aminotransferase

We used the AUTOSPAN® Liquid Gold AST kit (IFCC kinetic method; Arkray Healthcare Pvt. Ltd., India) to measure serum aspartate aminotransferase (AST) activity, following the manufacturer’s instructions. The assay was done at 37 °C with a 1 cm light path and a main wavelength of 340 nm. In short, 1,000 µL of working AST reagent was put into a cuvette and kept at 37 °C. Then, 100 µL of serum sample was added and mixed gently. After a 60-s wait, absorbance was measured every 30 s for three readings. We found the mean change in absorbance per minute (ΔA/min) and used the kinetic factor given by the manufacturer to express AST activity in U/L.

#### 2.12.3 Estimation of serum alkaline phosphatase

Serum alkaline phosphatase (ALP) activity was estimated using the TRUEchemie ALP Test Kit (SR) (IFCC method; REF No: ADX201, TRUchemie, India) according to the manufacturer’s instructions. The assay was performed at 37 °C with a primary wavelength of 405 nm in kinetic mode. Briefly, 1,000 µL of ALP reagent was added into a cuvette and pre-warmed to 37 °C, followed by the addition of 25 µL of serum sample and mixed gently. After an initial lag time of 30 s, absorbance was recorded at 30-s intervals up to 180 s. The mean change in absorbance per minute (ΔA/min) was calculated, and ALP activity was expressed in U/L using the kinetic factor provided by the manufacturer.

#### 2.12.4 Estimation of serum gamma-glutamyl transferase

We used the TRUEchemie GGT Test Kit (IFCC kinetic method; REF No: ADX191, TRUchemie, India) to measure serum Gamma-Glutamyl Transferase (GGT) activity, following the manufacturer’s instructions. The test was done at 37 °C with a primary wavelength of 405 nm in kinetic mode. To make the working reagent (4:1 ratio), 800 µL of Buffer Reagent (R1) was added to a cuvette that had been pre-warmed to 37 °C. Then, 200 µL of Substrate Reagent (R2) was added and mixed gently. Then, 100 µL of serum sample was added, mixed well, and the absorbance was measured after a 60-s delay. For 3 minutes, readings were taken every 30 s. We figured out the average change in absorbance per minute (ΔA/min) and used the kinetic factor given by the manufacturer to show GGT activity in U/L.

### 2.13 Measurement of pancreatic enzymes

#### 2.13.1 Estimation of serum alpha-amylase

Serum amylase activity was estimated using the TRUEchemie amylase Test Kit (Colorimetric method; REF No: AX261, TRUchemie, India) according to the manufacturer’s instructions. The assay was performed at 37 °C with The working reagent was pre-warmed to 37 °C, after which 1,000 µL of alpha-amylase reagent was added into a cuvette, followed by 25 µL of serum sample and mixed gently; absorbance was recorded at 405 nm after 30 s and subsequently at 30-s intervals for 90 s, the change in absorbance per minute (ΔA/min) was calculated, and alpha-amylase activity was determined using the formula: Amylase (U/L) = ΔA/min × 4,640.

#### 2.13.2 Estimation of serum lipase

Serum lipase activity was estimated using the TRUEchemie Lipase Test Kit (Colorimetric method; REF No: ADX306, TRUchemie, India) according to the manufacturer’s instructions. The assay was performed at 37 °C with a primary wavelength of 580 nm. Briefly, 800 µL of Lipase Reagent 1 (R1) was added to a cuvette followed by 16 µL of serum sample, mixed gently, and incubated for 3 min at 37 °C. Thereafter, 200 µL of Lipase Reagent 2 (R2) was added, mixed well, and absorbance was recorded at 580 nm after 60 s and again at 120 s. The change in absorbance (ΔA) was calculated, and lipase activity (U/L) was determined using the formula: Lipase (U/L) = (ΔAT/ΔAS) × concentration of calibrator, as per the manufacturer’s instructions.

### 2.14 Assessment of antioxidant and lipid peroxidation activities

The tissues were excised immediately after sacrifice, washed with in ice-cold normal saline to remove blood, blotted dry, and homogenized (10% w/v) in ice-cold phosphate-buffered saline (PBS,0.1M, pH 7.4) using a glass-Teflon homogenizer. The homogenates were centrifuged at 1,000 g for 15 min at 4 °C, and the resulting supernatants were collected and used for the estimation of oxidative stress markers (SOD, CAT, GSH and MDA) and total protein content. The total protein content of tissue homogenates was determined by the method of (Lowry et al., 1951) using bovine serum albumin (BSA) as the standard. The activities of SOD, CAT, as well as the levels of reduced glutathione (GSH), were normalized to the protein concentration and expressed as U/mg or nmol/g, respectively. MDA levels were expressed as nmol/g tissue according to the TBRAS assay protocol.

#### 2.14.1 Estimation of superoxide dismutase (SOD)

To assess the activity of superoxide dismutase (SOD), we combined 500 µL of tissue sample with 2 mL of 0.1 M Tris-HCl buffer (pH 8.2). To initiate the reaction, 500 µL of pyrogallol was incorporated into the 3 mL solution, which was subsequently diluted with distilled water. We quantified the absorbance at 420 nm and utilized the cessation of pyrogallol auto-oxidation to determine the activity of SOD (Marklund and Marklund, 1974).

#### 2.14.2 Estimation of catalase (CAT)

To find out how active catalase was, 1 mL of phosphate buffer was mixed with 100 µL of tissue sample, and then 400 µL of 0.2 M hydrogen peroxide (H_2_O_2_) was added. After 1 minute of incubation, 2 mL of dichromate-acetic acid reagent was added to stop the reaction. For 10 min, the mixture was heated in a boiling water bath, then cooled, and the absorbance was measured at 620 nm (Sinha, 1972).

#### 2.14.3 Estimation of reduced glutathione (GSH)

To find out how much reduced glutathione (GSH) was in the tissue sample, 100 µL was mixed with 2 mL of 5% TCA and then spun at 5,000 rpm for 15 min. We took 500 µL from the supernatant and mixed it with 1 mL of DTNB reagent. The volume was set to 3 mL, and the sample was left at room temperature for 5 minutes before measuring absorbance at 412 nm (Moron et al., 1979).

#### 2.14.4 Estimation of lipid peroxidation (MDA–TBARS method)

We used the thiobarbituric acid reactive substances (TBARS) method to measure malondialdehyde (MDA) levels to see how much lipid peroxidation had happened. In short, 250 µL of a tissue sample was combined with 1.5 mL of 20% TCA and 1.5 mL of thiobarbituric acid (TBA) reagent. For 15 min, the mixture was heated in a boiling water bath and then cooled. After that, 3 mL of n-butanol was added and mixed well. The absorbance of the organic layer was then measured at 532 nm (Ohkawa et al., 1979).

### 2.15 Histopathologic examination

Tissues extracted from experimental rats (pancreas and liver) were thoroughly flushed with phosphate buffer (0.1 M) prior to fixation with neutral buffered formalin 10%. The specimens were subsequently processed by dehydrating through a series of ethanol gradations and then clearing with xylene and finally embedded in paraffin. Subsequently, a microtome was employed to section the paraffin-embedded tissue blocks at 5 microns thick. Hematoxylin and eosin (H&E) staining was performed on the tissue sections and observe through a Zeiss (India) compound microscope to determine any identified pathological alteration. A semi-quantitative scoring methodology was utilized to determine the degree of change objectively. All tissue sections were processed simultaneously using identical fixation, embedding, sectioning, and haematoxylin-eosin (H&E) staining protocols. Histological images were acquired under standardized microscope settings, including magnification, illumination, and exposure time, to ensure reliable comparison among experimental groups. Histopathological examination and scoring were performed by an experienced pathologist who was blinded to the treatment groups to minimize the observational bias.

### 2.16 Immunohistochemical staining

The expression of TNF-α and IL-1β in paraffin-embedded sections of liver and pancreas had been determined using immunohistochemical properties. The paraffin was removed from the slices (5 µm) using xylene followed by rehydration using progressively increasing ethanol solutions. The antigen retrieval process was accomplished using citrate buffer (10 mM, pH 6.0) at 95 °C–98 °C for 15 min and the buffer was then allowed to cool to room temperature. Endogenous peroxidase action was inhibited using the incubation of the sections with 3% hydrogen peroxide for 10 min. The next day, following the incubation period, the sections were placed into a controlled humidity chamber at 4 °C overnight using the following primary antibodies: rabbit polyclonal anti-TNF-α (Abcam, ab6671; 1:200) and rabbit polyclonal anti-IL-1β (Abcam, ab9722; 1:200). Controls for this experiment included sections that had not received the primary antibodies (negative control) and those that were treated with isotype matched rabbit IgG at the same concentration (negative control) and inflamed tissue sections were used as positive control. Following the washing procedure, the sections were placed into an incubator at room temperature for 1 hour using HRP conjugated anti-rabbit secondary antibody (1:500; (Abcam, ab6721; 1:500) as a secondary antibody. The antibodies were chosen based on the manufacturer’s recommendations and prior use in rat tissue immunohistochemistry studies. The sections were viewed using the chromogen 3,3′-diaminobenzidine (DAB) to detect immunoreactivity and then stained with haematoxylin. Slides were cleaned, fixed and observed under a light microscope. Sections obtained from the VPA-treated group, which exhibited pronounced inflammatory changes and strong TNF-α/IL-1 immunoreactivity, were used as positive controls for immunohistochemical staining. Negative controls were processed identically except that the primary antibody was omitted. The quantitative measurement of immunohistochemical staining was performed using ImageJ software. The percentage of the positive area of TNF-α/IL-1β was used to quantify the expression levels.

All images were captured at the same magnification level of 10×; therefore, as part of the methodology utilised during quantitative measurements, called colour deconvolution (H-DAB), we were able to differentiate DAB staining, from the background hematoxylin counterstain, by converting the images into 8-bit grey scale. Immunohistochemical staining was quantified using ImageJ software by measuring the DAB-positive staining area under a fixed threshold applied uniformly by across all images. As the present study was designed as a preclinical animal investigation, quantitative image analysis was preferred over semi-quantitative clinical scoring system such as H-score or Allred score to provide objective and reproducible assessment of protein expression.

### 2.17 Statistical analysis

Normality of the data distribution was assessed using a Shapiro-Wilk test, and homogeneity of variance was evaluated using the Brown-Forsythe test prior to statistical analysis. A one-way ANOVA was run to evaluate statistical differences between six different groups, and Tukey’s *post hoc* analysis was performed to assess pairwise differences. The p-value cut-off for statistical significance was <0.05. The data were analysed using GraphPad Prism v10.6.1 and presented both in tabular and graphic form. All statistical comparisons were planned *a priori* based on the primary study hypothesis evaluating the protective effects of ALRE against VPA-induced hepatopancreatic toxicity.

## 3 Results

### 3.1 Extraction yield, phytochemical characterization and phytochemical activity of *Arctium lappa* root extract

#### 3.1.1 Percentage yield (%)

The yields (%) of ALRE were very diverse across all the solvent systems tested. The highest yield occurred from the methanol: water (50:50 v/v) extract of ALRE (12.80% ± 0.15%), followed by the methanolic extract (10.23% ± 0.12%). Yields of acetone and ethanol were moderate (6.70% ± 0.23%), while the aqueous extract had the lowest yield (4.27% ± 0.09%). This shows that both hydroalcoholic and methanolic solvent systems had superior extraction capability compared to their respective organic solvents and water in extracting bioactive compounds from the *A. lappa* root. These results are shown in Figure 2a.

**FIGURE 2:**
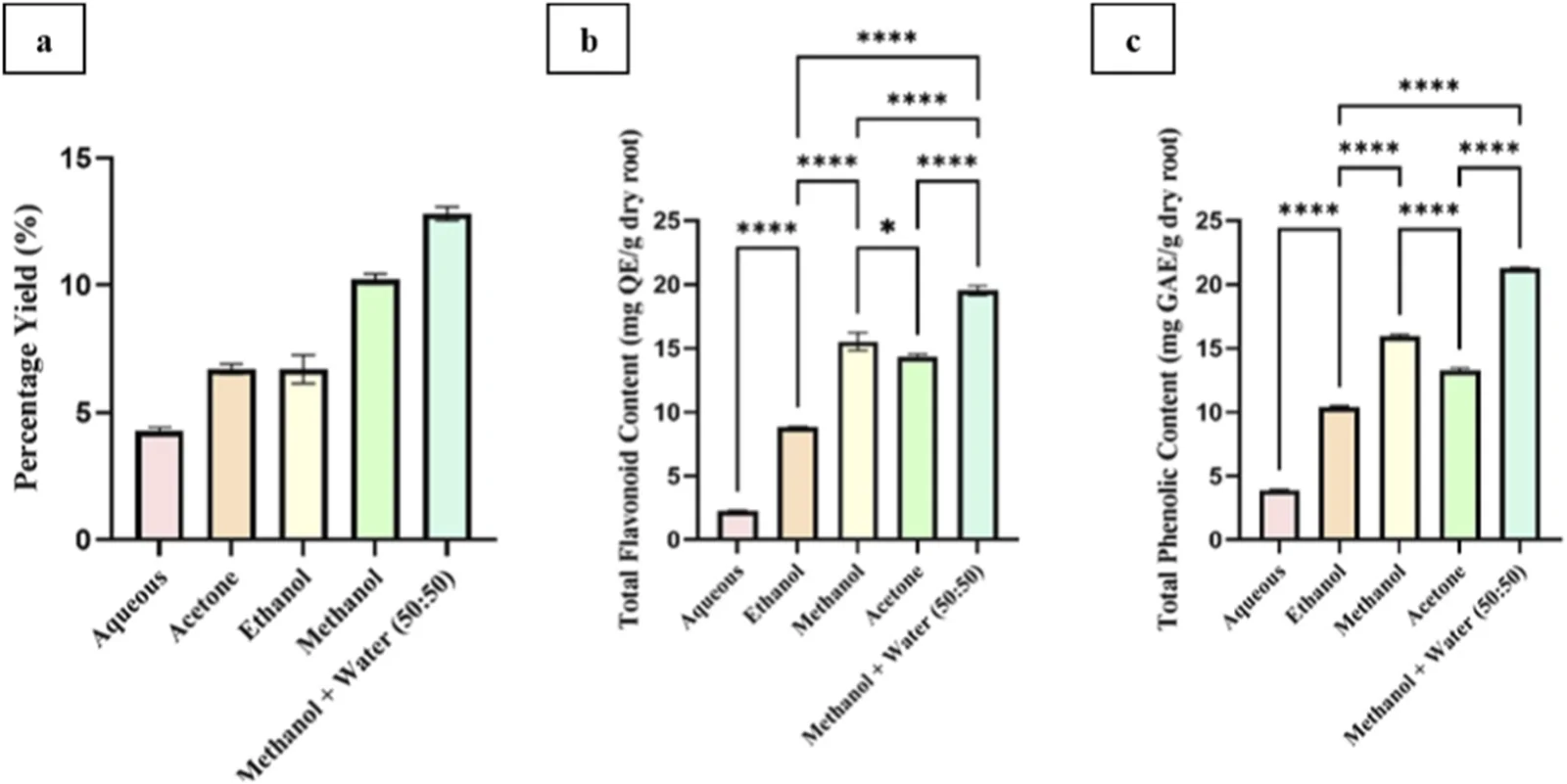
Extraction yields and phytochemical content of ALRE **(a)** The percentage yield (%) obtained from ALRE using a different solvent (aqueous, acetone, ethanol, methanol and 50:50 Methanol-Water) as well as the total flavonoid content (mg of quercetin equivalent per gram of dry root) in **(b,c)** total phenol content (mg of gallic acid equivalent per gram of dry root) are displayed in the graphs. The means and standard errors of means (+/− SEM) are shown. Error was calculated from three experiments. Each experiment was analysed statistically by one-way ANOVA with Tukey’s Multiple Comparisons post-test to determine whether groups differed significantly from one another. Asterisks indicate statistically significant differences between groups (*p < 0.05, ***p < 0.001, ****p < 0.0001).

#### 3.1.2 GC–MS analysis

GC-MS analysis of ALRE was performed using a PerkinElmer Clarus 680 gas chromatograph coupled with a Clarus 600 mass spectrometer. The chromatogram revealed several well-resolved peaks corresponding to volatile and semi-volatile phytoconstituents, indicating efficient separation of the sample components (Supplementary Material 1). Compound identities were tentatively assigned by comparing the obtained mass spectra with the NIST library database. Table 2 presents the top NIST library match (highly reverse match (REV) score) for six selected major peaks. All reported compounds should therefore be considered tentative identifications based on spectral library matching.

**TABLE 2:** Tentatively identified Compounds of ALRE based on GC-MS and NIST Library Matching.

S. No.	Retention Time	Compound name	Molecular weight (g/mol)	Molecular Formula	Peak area (%)	PubChem CID
1	11.262	O-Methylisourea	74	C_2_H_6_ON_2_	15.63	75544
2	13.713	1(2H)-Napthalenone,3,4-dihydro-6-methoxy	176	C_11_H_12_O_2_	17.41	19392556
3	17.154	3-Thiophenamine, tetrahydro-N-methyl-N-nitroso-1,1-dioxide	178	C_5_H_10_O_3_N_2_S	10.15	25810
4	18.670	Z, Z-3,11-Octadecadien-1-ol acetate	308.5	C_20_H_36_O_2_	1.19	5363502
5	18.930	DL-Homoserine	119	C_4_H_9_NO_3_	9.28	779
6	26.493	3,4-Epoxytetrahydrothiophene-1,1-dioxide	134	C_4_H_6_O_3_S	3.72	20597

#### 3.1.3 HPLC profiling of *Arctium lappa* root extract

The HPLC chromatographic profile of hydroalcoholic extract of *Arctium lappa* roots using a detection wavelength of 280 nm revealed the presence of 17 peaks indicating the presence of numerous phenolics and lignan constituents as mentioned in Table 3. The highest peak was observed at the retention time of 5.013 min with 69.94% of the area of the whole chromatogram and hence belongs to the major component of the extract (Al-Shammaa et al., 2017). The complete HPLC chromatogram is presented in the Supplementary Material 1. Through comparison with the already published data on HPLC chromatograms, the major peak can currently be tentatively identified as arctiin that is the main bioactive lignan and the marker compound of *A. lappa.* The peaks observed at the retention times 6.305, 6.817, 7.379, 7.939, 9.450, and 9.751 min may represent several other well-known phytochemicals such as arctigenin, chlorogenic acid, caffeic acid, and some derivatives of dicaffeoylquinic acids as well as some lappaol-type lignans described in literature on the roots of *A. lappa* published using HPLC-DAD and LC-MS (Liu et al., 2005; Lin and Harnly, 2008; Ferracane et al., 2010). Given that the retention time will vary depending on the experimental conditions such as stationary phase, composition of the mobile phase, flowrate, and wavelength of detection, the identified compounds must be considered preliminary, and their identification should be confirmed using standard references.

**TABLE 3:** HPLC peak profile of *Arctium lappa* root hydroalcoholic extract detected at 280 nm.

Peak	Ret. Time	Area	Height	Area %	Height %
1	3.200	4,328	187	0.011	0.017
2	5.013	27181650	638554	69.941	58.654
3	6.305	6313948	205519	16.246	18.878
4	6.817	1275736	65602	3.283	6.026
5	7.379	1576700	53847	4.057	4.946
6	7.939	1707360	84907	4.393	7.799
7	8.951	11896	1,186	0.031	0.109
8	9.450	261116	13459	0.672	1.236
9	9.751	109879	8,593	0.283	0.789
10	10.203	44137	3,101	0.114	0.285
11	12.038	199817	8,159	0.514	0.749
12	13.190	56806	1844	0.146	0.169
13	14.094	20002	863	0.051	0.079
14	16.893	42476	1,165	0.109	0.107
15	19.757	13474	410	0.035	0.038
16	20.660	25225	735	0.065	0.068
17	24.797	19076	538	0.049	0.0490
Total	​	38863627	1088671	100.000	100.000

#### 3.1.4 Estimation of total flavonoid content

The TFC of ALRE was significantly different across the different extraction solvents. The flavonoid concentration was greatest in the methanol-water (50:50, v/v) extract (19.93 mg QE/g dry root) followed by the methanolic extract (15.05 mg QE/g dry root), the ethanolic extract (8.81 mg QE/g dry root) and the aqueous extract (2.28 mg QE/g dry root) (Figure 2b).

#### 3.1.5 Estimation of total phenolic content

The TPC of ALRE was greatest in the methanol-water (50:50) extract (21.36 mg GAE/g dry root), followed by the methanolic extract (15.83 mg GAE/g dry root), the acetone extract (13.25 mg GAE/g dry root), the ethanolic extract (10.35 mg GAE/g dry root) and the aqueous extract (3.99 mg GAE/g dry root) (Figure 2c).

### 3.2 *In vitro* antioxidant activity of *Arctium lappa* root extract

#### 3.2.1 DPPH radical scavenging activity

The DPPH radical scavenging experiment was used to assess the antioxidant capacity of the aqueous, acetone, ethanol, methanol, and hydroalcoholic extracts of *Arctium lappa* root; the IC50 values are shown in Table 4. The hydroalcoholic (methanol: water) extract showed the best antioxidant potential and the strongest DPPH radical scavenging activity among the studied extracts, with the lowest IC50 value of 370.70 μg/mL as shown in Figure 3a. Methanolic extract (414.23 μg/mL), acetone extract (436.07 μg/mL), aqueous extract (463.06 μg/mL), and ethanolic extract (468.90 μg/mL) came next. When compared to all plant extracts, the conventional antioxidant BHT showed improved radical scavenging action with a much lower IC50 value of 310.11 μg/mL. The hydroalcoholic extract was chosen for further phytochemical and biological studies because it had the highest antioxidant potential of all the studied extracts.

**TABLE 4:** IC50 values of DPPH and ABTS scavenging assays.

Extract	DPPH IC50 (µg/mL)	ABTS IC50 (µg/mL)
Aqueous	463.06	477.41
Acetone	436.07	456.65
Ethanol	468.90	418.33
Methanol	414.23	370.43
Methanol: Water (50:50)	370.70	441.87
Standard	310.11	334.31

**FIGURE 3:**
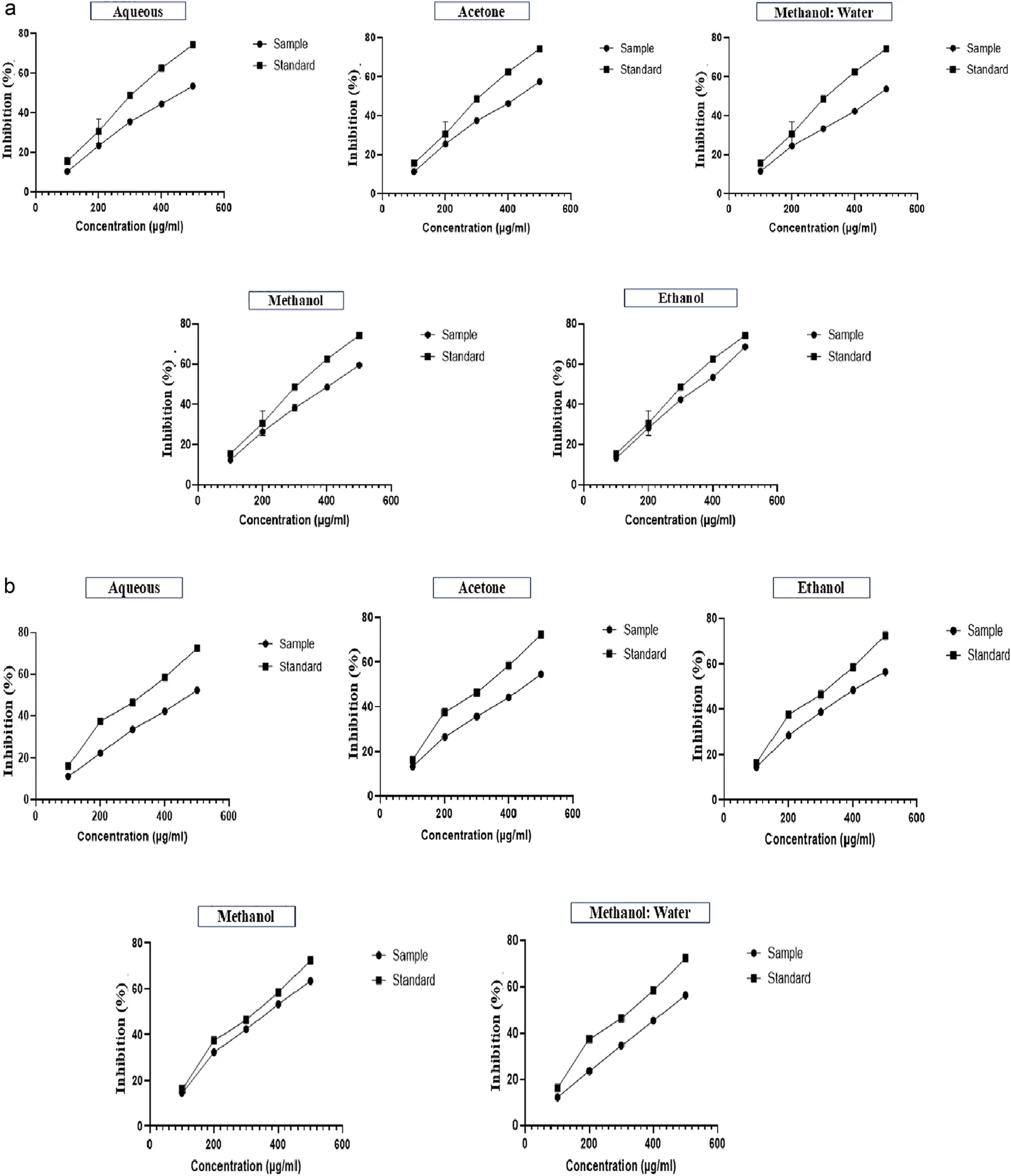
**(a)** Aqueous, acetone, methanol: water (50:50), methanol, and ethanol extracts of *Arctium lappa* root were tested for their ability to scavenge DPPH radicals in comparison to the conventional antioxidant BHT. For all extracts and the standard, the percentage inhibition of DPPH radicals rose in a concentration-dependent manner (100–500 μg/mL). The data are displayed as mean ± SEM (n = 3). Among the studied extracts, the hydroalcoholic (methanol: water) extract had the highest DPPH radical scavenging activity, while BHT had better antioxidant activity than any other extract. **(b)** Aqueous, acetone, ethanol, methanol, and hydroalcoholic (methanol: water, 50:50) extracts of *Arctium lappa* root were tested for their ability to scavenge ABTS radicals in comparison to the common antioxidant vitamin C. For all extracts and the standard, the percentage inhibition of ABTS radicals rose in a concentration-dependent manner (100–500 μg/mL). Mean ± SEM (n = 3) is used to express the data. Vitamin C demonstrated higher antioxidant activity than any of the studied extracts, but the methanolic extract had the highest ABTS radical scavenging activity.

#### 3.2.2 ABTS^+^ radical scavenging activity

The ABTS radical scavenging assay was used to assess the antioxidant activity of the aqueous, acetone, ethanol, methanol, and hydroalcoholic (methanol: water, 50:50) extracts of *Arctium lappa* root as shown in Figure 3b. The IC50 values are shown in Table 4. The methanolic extract had the best antioxidant capacity among the studied extracts, exhibiting the highest ABTS radical scavenging activity and the lowest IC50 value of 370.43 μg/mL. The ethanolic extract (418.33 μg/mL), hydroalcoholic (methanol: water) extract (441.87 μg/mL), acetone extract (456.65 μg/mL), and aqueous extract (477.41 μg/mL) came next. The standard antioxidant, vitamin C, exhibited higher radical scavenging activity than all the studied extracts, with a lower IC50 value of 334.31 μg/mL. Among the *A. lappa* root extracts tested in the ABTS assay, the methanolic extract showed the highest overall antioxidant capacity.

### 3.3 Cell viability assay

Using the MTT test, the cytotoxic capability of the hydroalcoholic *Arctium lappa* root extract was assessed against Hep3B and PANC-1 cell lines throughout a concentration range of 25–1,250 μg/mL (Figure 4). At concentrations between 25 and 500 μg/mL, the extract showed very little cytotoxicity, and both cell lines-maintained cell viability above 90%. Higher doses (750–1,250 μg/mL) showed a concentration-dependent decrease in cell viability, suggesting greater cytotoxicity as extract concentration increased. The hydroalcoholic extract exhibited comparatively low cytotoxic activity against both cancer cell lines, as evidenced by the calculated IC50 value of 1,184.46 μg/mL for the Hep3B cell line and 1,234.11 μg/mL for the PANC-1 cell line. Cell viability was significantly lower in the positive control treatment than in the negative control (p < 0.05). Overall, the findings support the hydroalcoholic *A. lappa* root extract’s safety for additional pharmacological testing by showing minimal cytotoxicity against Hep3B and PANC-1 cells within the studied dose range.

**FIGURE 4:**
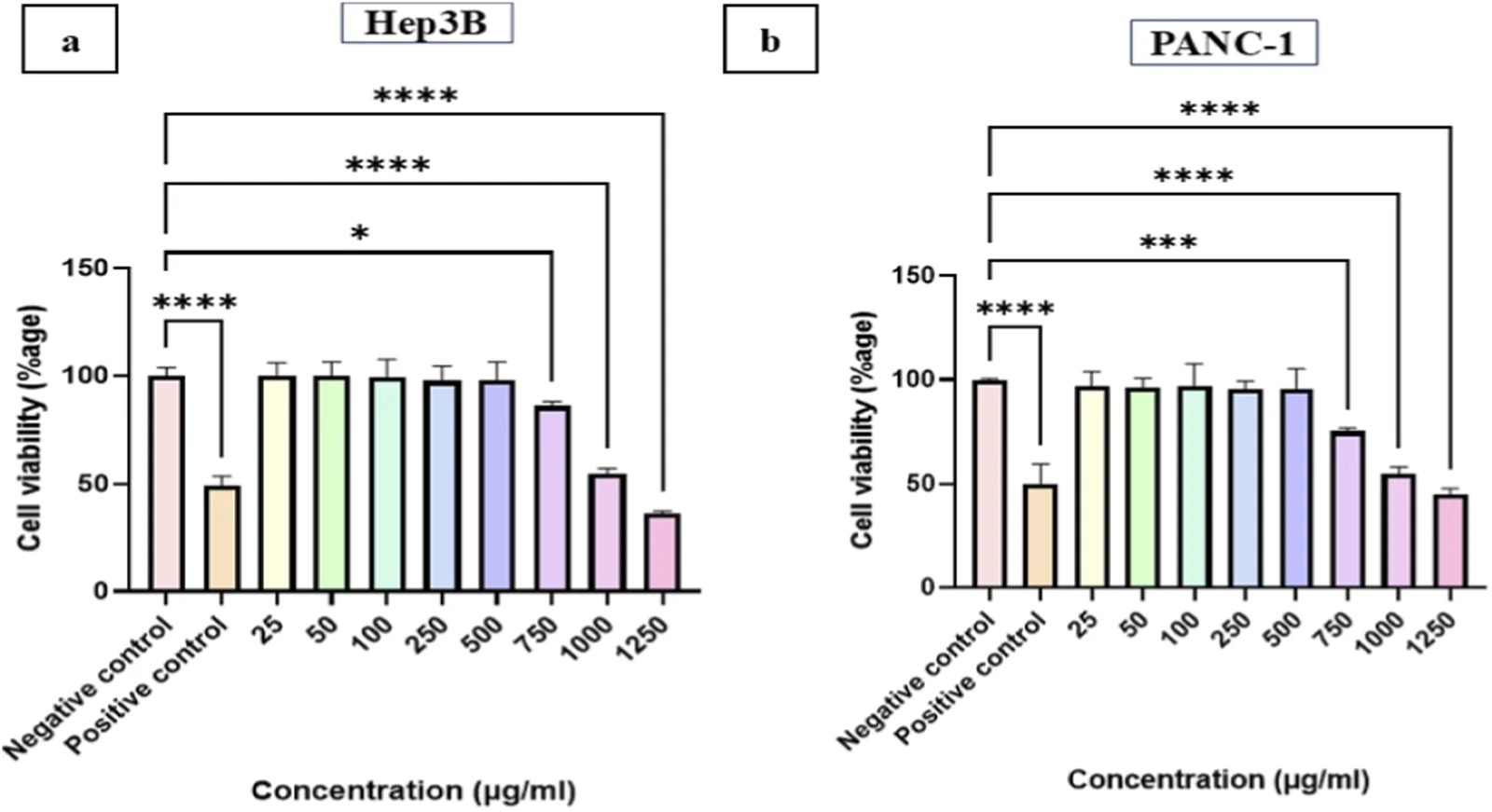
The cytotoxic properties of hydroalcoholic extract from *Arctium lappa* root on **(a)** Hep3B and **(b)** PANC-1 cell lines were evaluated using MTT assay post 24 h treatment. The results are shown as mean ± SEM (n = 3). One way ANOVA along with Tukey’s multiple comparison test was used for the statistical analysis (p < 0.05). The calculated IC50 value for both cell line is 1,184.46 μg/mL for Hep3B and 1,234.11 μg/mL for PANC-1, which denotes low cytotoxic effect of the extract against both the cell lines.

### 3.4 Cell viability assay using normal hepatic (THLE-2) and pancreatic (H6C7) cell lines

The MTT test was used to assess the extract’s cytotoxic potential in normal human liver epithelial (THLE-2) and normal human pancreatic duct epithelial (H6C7) cell lines. Cell viability was reduced by the extract in a concentration-dependent manner, but significant viability was preserved across the measured concentration range. Cell viabilities in THLE-2 cells treated with the extract at doses of 250, 500, 750, 1,000, and 1,250 μg/mL were 94.23%, 84.89%, 74.45%, 56.23%, and 38.67%, respectively. Cell viability was 47.30% in the positive control (doxorubicin) and 98.58% in the negative control. An IC50 value of 1,084.92 μg/mL was obtained by nonlinear regression analysis, showing little cytotoxicity against normal liver cells.

Similarly, at doses of 250, 500, 750, 1,000, and 1,250 μg/mL, the extract showed cell viabilities of 93.87%, 80.69%, 69.50%, 58.49%, and 46.21% in H6C7 cells. Cell viabilities for the positive and negative controls were 45.82% and 96.30%, respectively. With an IC50 value of 1,174.62 μg/mL, it was determined to have no cytotoxic effect on normal pancreatic cells. Overall, the extract has a good safety profile and shows minimal toxicity toward normal hepatic and pancreatic cells under the studied conditions, as evidenced by the high IC50 values seen in both THLE-2 and H6C7 cell lines as shown in Figure 5.

**FIGURE 5:**
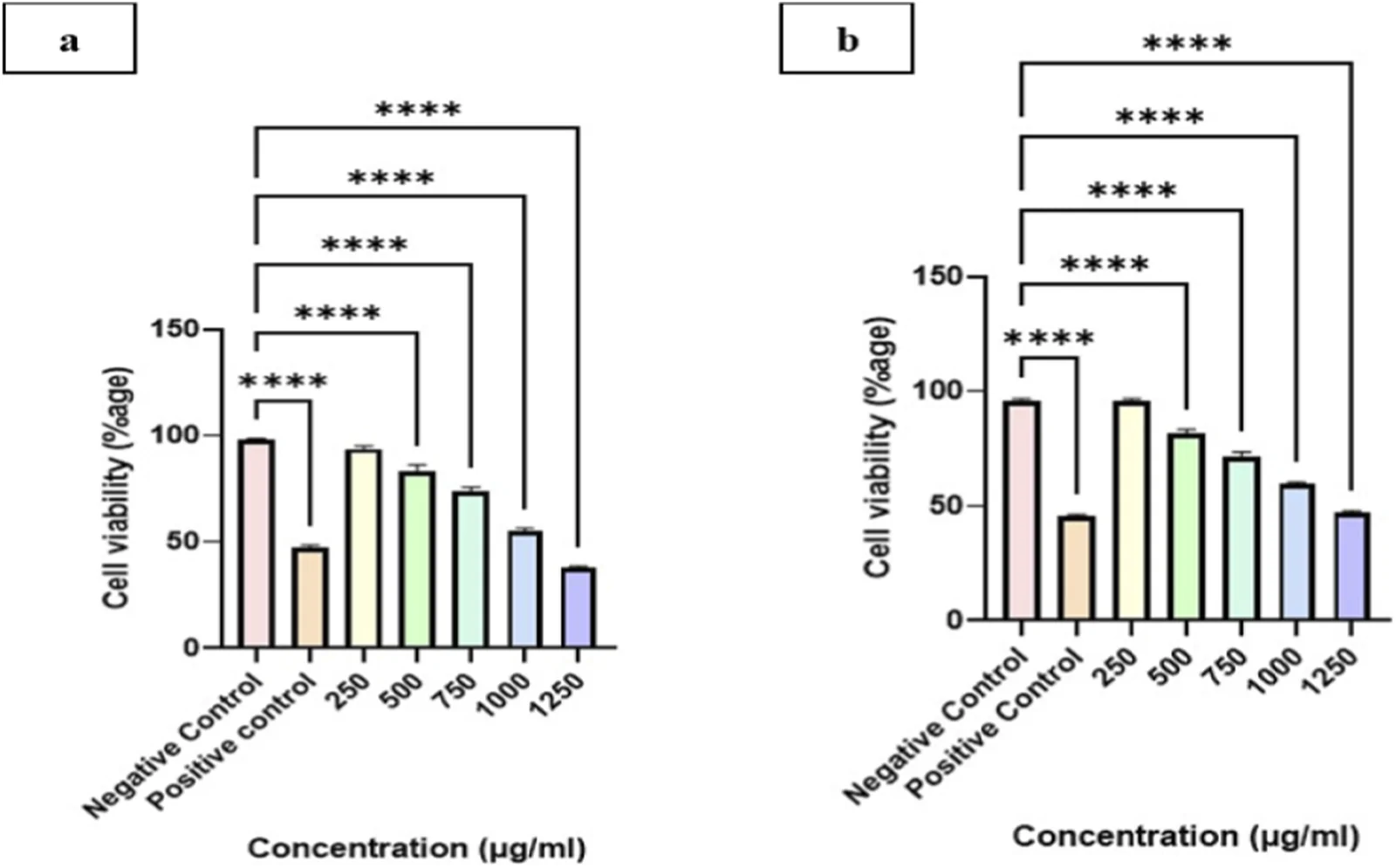
The MTT assay was used to assess the extract’s impact on the viability of THLE-2 and H6C7 cells. The percentage of cell viability in relation to the negative control is used to express the data. With IC50 values of 1,084.92 μg/mL (THLE-2) **(a)** and 1,174.62 μg/mL (H6C7) **(b)**, the extract demonstrated concentration-dependent cytotoxicity, showing modest toxicity toward normal cells.

### 3.5 Effect of ALRE on body weight, liver index and pancreatic index

When compared to the control group, VPA administration considerably raised body weight, liver index, and pancreatic index. These changes were considerably improved by treatment with ALRE at both low and high dosages, with the high dose exhibiting stronger protective benefits. The VPA-induced alterations were likewise considerably lessened by L-carnitine therapy as shown in Figure 6. Furthermore, ALRE by itself had no negative effects on these parameters, demonstrating its safety. These results imply that ALRE successfully guards against hepatic and pancreatic damage caused by VPA.

**FIGURE 6:**
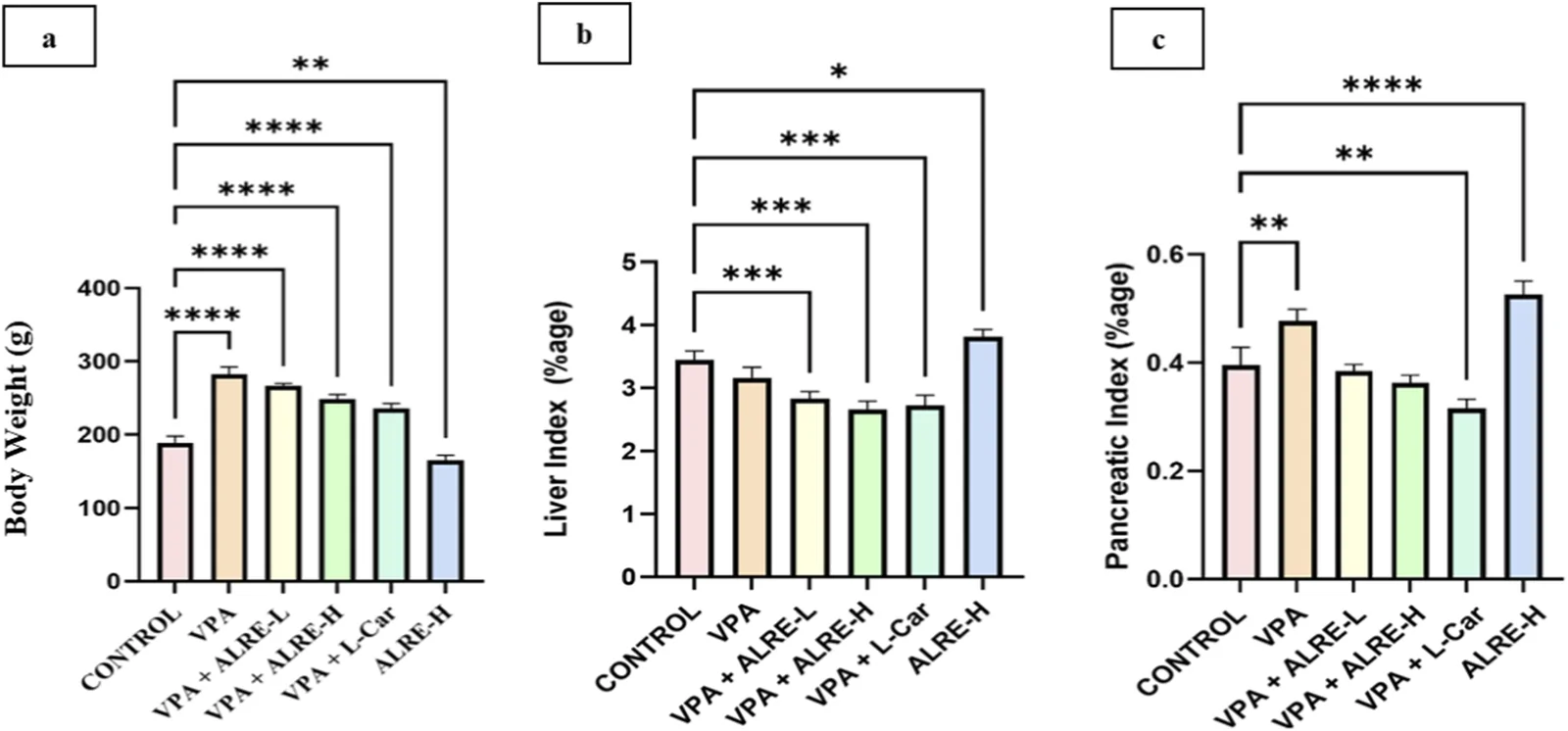
Shows the impact of ALRE on **(a)** body weight, **(b)** liver index, and **(c)** pancreatic index in rats with pancreatitis and hepatotoxicity caused by VPA. When compared to the control group, VPA administration considerably increased body weight, liver index, and pancreatic index. These changes were attenuated by treatment with L-carnitine and ALRE (300 and 500 mg/kg), with the higher dose of ALRE showing stronger protective effects. Data are expressed as mean ± SEM (n = 6). *p* < 0.05, p < 0.01, *p < 0.001, and p < 0.0001.

### 3.6 Hepatocellular enzyme levels

The administration of VPA creates an enormous increase in the serum hepatic enzyme levels (ALT, AST, ALP, GGT) compared to controls indicating that VPA causes significant hepatotoxicity (Figure 7). Co-Administration of hydroalcoholic root extract of *Arctium lappa* at doses of 300 mg/kg and 500 mg/kg results in a dose-dependent reduction in ALT levels, demonstrating the hepatoprotective potential of hydroalcoholic root extract of the *A. lappa*. Similar reductions in AST levels were also observed with both doses of root extract but were particularly pronounced when using the 500 mg/kg dose. The reference hepatoprotective drug used in this study L-carnitine (300 mg/kg) also reduced the VPA-induced increase in ALT and AST levels, supporting its hepatoprotective reference role as mentioned in Table 5.

**FIGURE 7:**
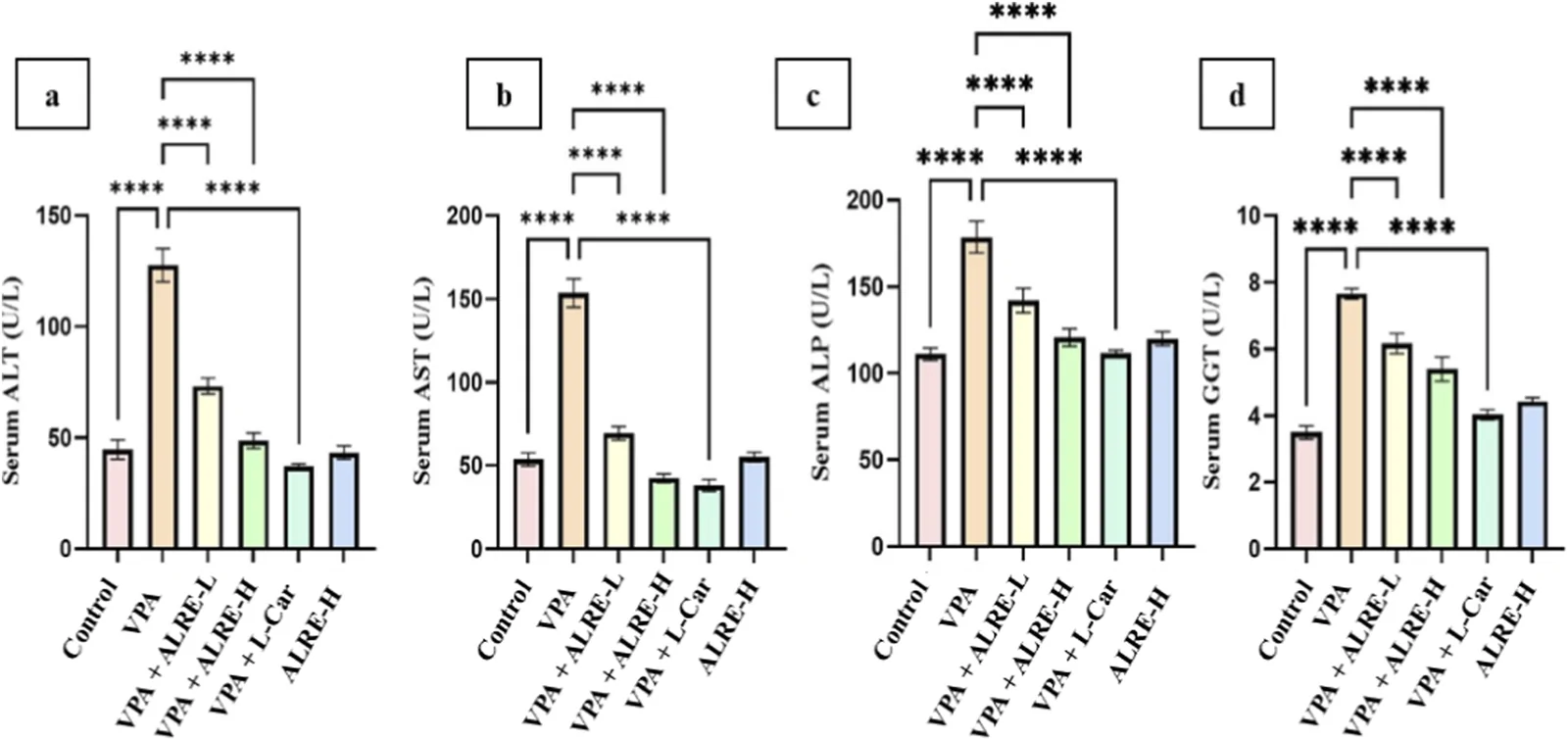
Effects of ALRE and carnitine on serum liver enzyme levels in VPA-treated rats. The levels of ALT **(a)**, AST **(b)**, ALP **(c)**, and GGT **(d)** were measured following treatment with either VPA or ALRE and L-Carnitine and compared with control animals that received no treatment. The data presented are the mean ± SEM of the four groups. One-way ANOVA followed by Tukey’s multiple comparison procedure was used for statistical analyses of the data. The statistical significance of differences between groups is indicated by asterisks, where * indicates p < 0.05, ** indicates p < 0.01, *** indicates p < 0.001, and **** indicates p < 0.0001. With η^2^ values of 0.9875 (ALT), 0.9904 (AST), 0.9644 (ALP), and 0.9822 (GGT), effect size analysis using eta-squared (η^2^) showed very large treatment effects for all liver function parameters. This means that the treatment groups explained 98.75%, 99.04%, 96.44%, and 98.22% of the total variance in these parameters, respectively.

**TABLE 5:** Effect of ALRE on hepatic and pancreatic function.

Groups	ALT (U/L)	AST (U/L)	ALP (U/L)	GGT (U/L)	Serum amylase (U/L)	Serum lipase (U/L)
Control	44.67 ± 2.60	53.67 ± 2.33	111.00 ± 2.08	3.50 ± 0.12	15.67 ± 1.20	17.67 ± 1.45
VPA	127.67 ± 4.34	153.67 ± 4.91	178.67 ± 5.21	7.67 ± 0.09	47.33 ± 2.91	49.00 ± 3.79
VPA + ALRE-L	73.33 ± 2.03	69.67 ± 2.40	142.00 ± 4.04	6.17 ± 0.18	35.33 ± 1.76	36.00 ± 1.15
VPA + ALRE-H	48.67 ± 2.03	42.33 ± 1.45	120.67 ± 2.96	5.40 ± 0.21	28.67 ± 2.40	30.00 ± 1.15
VPA + L-Car	37.00 ± 0.58	38.00 ± 2.08	111.67 ± 0.88	4.03 ± 0.09	26.67 ± 1.76	25.67 ± 2.33
ALRE-H	43.33 ± 1.77	55.00 ± 1.73	120.00 ± 2.31	4.43 ± 0.07	21.00 ± 2.08	22.33 ± 1.45

In addition, ALP and GGT levels, which were significantly elevated by VPA-induced toxicity and were significantly reduced when animals received treatment with ALRE and L-carnitine indicating that treated animals had recovered their hepatic function and membrane integrity. Of all treatments investigated in this study, the hydroalcoholic root extract of *Arctium lappa* at 500 mg/kg had the greatest hepatoprotective effect, closely approaching the effectiveness of the reference drug L-carnitine, illustrating the therapeutic potential of hydroalcoholic root extract of *A. lappa* for treatment of VPA-induced hepatotoxicity.

### 3.7 Pancreatic enzyme levels

Figure 8 illustrates the effect of ALRE and L-Carnitine on serum amylase and lipase levels, as well as the effect of an ALRE and L-Carnitine co-administration on the increased serum amylase and lipase levels caused by VPA-induced pancreatic toxicity. The increased serum amylase and lipase levels that resulted from the administration of VPA were found to be statistically significant (p < 0.05) in comparison to those of the control group and thus were indicative of the VPA-induced pancreatic dysfunction. Furthermore, when ALRE was co-administered with either 300 mg/kg or 500 mg/kg of VPA, the levels of amylase and lipase were significantly lower than those of the VPA-treated animals, which indicates that ALRE plays a protective role in maintaining pancreatic function. The co-administration of L-Carnitine (300 mg/kg) also demonstrated a significant decrease in the enzyme levels consistent with the known cytoprotective effects of L-Carnitine. It should be noted that treatment of ALRE at 500 mg/kg alone did not produce a significant effect on serum amylase and lipase levels when compared to the control group but did cause a significant reduction in serum amylase and lipase levels in the VPA-treated animals. This indicates that both ALRE and L-Carnitine protect against VPA-induced pancreatic toxicity, with ALRE at the highest dose demonstrating the greatest level of protection.

**FIGURE 8:**
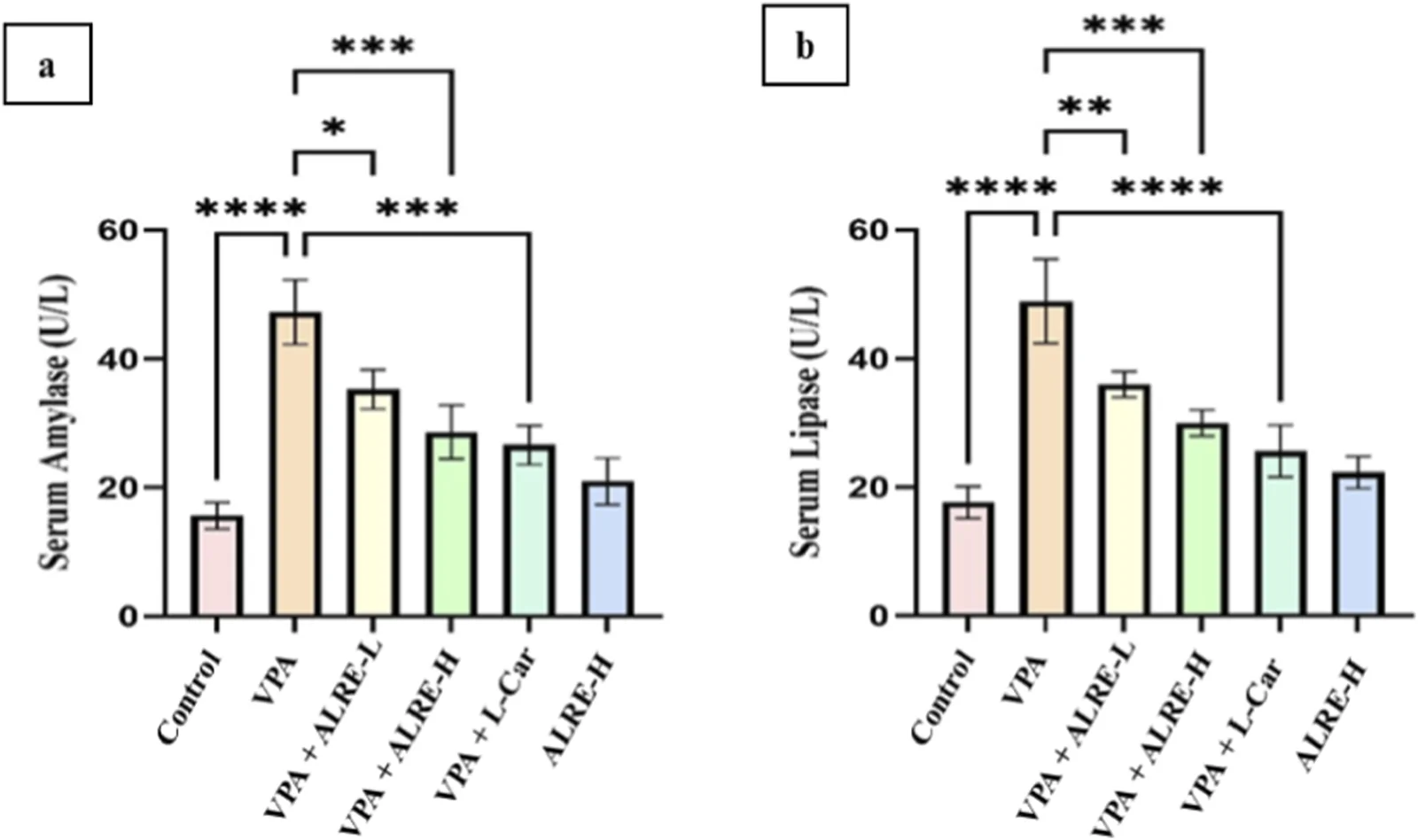
Effects of ALRE and carnitine on serum pancreatic enzyme levels in VPA-treated rats. **(a)** Serum amylase and **(b)** serum lipase levels in control, VPA-treated, and treatment groups. Data are presented as mean ± SEM. Statistical analysis was performed using one-way ANOVA followed by Tukey’s multiple comparisons test. Statistical significance is indicated by asterisks as follows: *p < 0.05, **p < 0.01, ***p < 0.001, ****p < 0.0001. Eta-squared (η^2^) effect size analysis showed very large treatment effects for both pancreatic enzymes, with η^2^ values of 0.9224 for amylase and 0.9216 for lipase, meaning that the treatment groups accounted for 92.24% and 92.16% of the total variance in serum amylase and lipase levels, respectively.

### 3.8 Liver antioxidant enzyme activity and lipid peroxidation

The quantification of oxidative stress status in the liver was conducted via evaluation of levels of antioxidant enzyme activity (SOD; CAT; GSH) and measurement of MDA levels (lipid peroxidation) in liver tissue. For the VPA-treated cohort, levels of antioxidant enzymes (SOD, CAT, GSH) were significantly decreased relative to the control group (p < 0.05), while levels of MDA (lipid peroxidation) were significantly elevated indicating the robust effect of VPA in generating oxidative stress and hepatic injury. The concurrent administration of ALRE at 300 mg/kg and 500 mg/kg resulted in a significant decrease in oxidative damage, as evidenced by a significant increase in SOD; CAT; GSH relative to those animals treated only with VPA. Additionally, it was demonstrated that co-treatment with ALRE resulted in a marked reduction in MDA levels, suggesting a significant reduction in lipid peroxidation, and improvement in hepatic redox status. While both ALRE doses improved antioxidant enzyme activity, the 500 mg/kg treated cohort demonstrated the most significant re-established antioxidant capabilities, with levels nearing normal ranges. Additionally, L-carnitine (300 mg/kg) was utilized as a reference antioxidant agent with similar hepatoprotective benefits through amelioration of both antioxidant enzyme levels and decreased MDA concentrations to that of ALRE-treated animals. Notably, the ALRE-alone (500 mg/kg) group did not exhibit any deleterious effects, maintaining normal antioxidant and MDA levels, thus highlighting the safety and antioxidant potency of the extract as shown in Figure 9.

**FIGURE 9:**
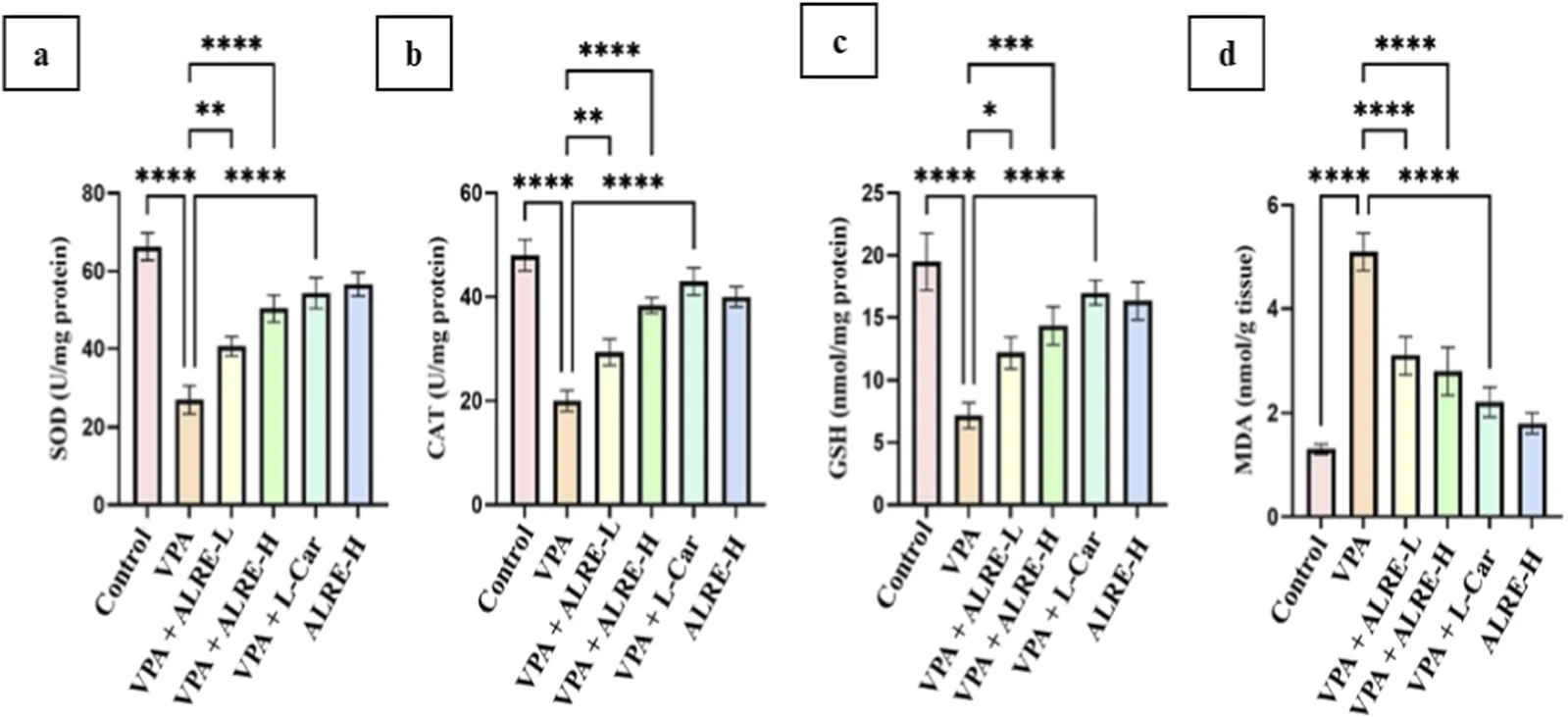
Effects of ALRE and L-carnitine on oxidative stress and antioxidant defence markers in hepatic tissue of VPA-treated rats. **(a)** superoxide dismutase (SOD), **(b)** catalase (CAT), **(c)** reduced glutathione (GSH), and **(d)** malondialdehyde (MDA) levels in control, VPA-treated, and treatment groups. Data are presented as mean ± SEM. Statistical analysis was performed using one-way ANOVA followed by Tukey’s multiple comparisons test. Statistical significance is indicated by asterisks as follows: *p < 0.05, **p < 0.01, ***p < 0.001, ****p < 0.0001. With η^2^ values of 0.9593 (CAT), 0.9530 (SOD), 0.9120 (GSH), and 0.9571 (MDA), effect size analysis using eta-squared (η^2^) showed very large treatment effects for all oxidative stress parameters. This means that the treatment groups explained 95.93%, 95.30%, 91.20%, and 95.71% of the total variance in these biomarkers, respectively.

### 3.9 Antioxidant and lipid peroxidation activity in pancreas

To evaluate the protective effects of ALRE and L-Carnitine against the damage caused by VPA-induced oxidative stress, the activity of the pancreatic antioxidant enzymes as well as the oxidative stress markers have been evaluated. There was a significant decrease in the post-treatment levels of SOD, CAT and GSH in the VPA-treated group when compared to the control. This indicates that the antioxidant defence system has been severely impaired by the VPA treatment. In addition, there was an increase in the levels of MDA in the VPA-treated group indicating greater levels of lipid peroxidation and oxidative stress occurring within the pancreatic tissues. Co-administration of ALRE at doses of both 300 and 500 mg/kg was able to significantly restore the levels of SOD, CAT and GSH, while simultaneously reducing the levels of MDA, with the 500 mg/kg dose exhibiting significantly greater effects. The administration of VPA in combination with carnitine (300 mg/kg) was also able to significantly reduce the oxidative stress markers although the level of reduction was less than that observed with ALRE at the 500 mg/kg dose. ALRE alone (500 mg/kg) produced a significant increase in levels of antioxidant enzymes and a significant decrease in MDA; therefore, it is evident that ALRE does display intrinsic antioxidant capacity. The data is presented in Figure 10.

**FIGURE 10:**
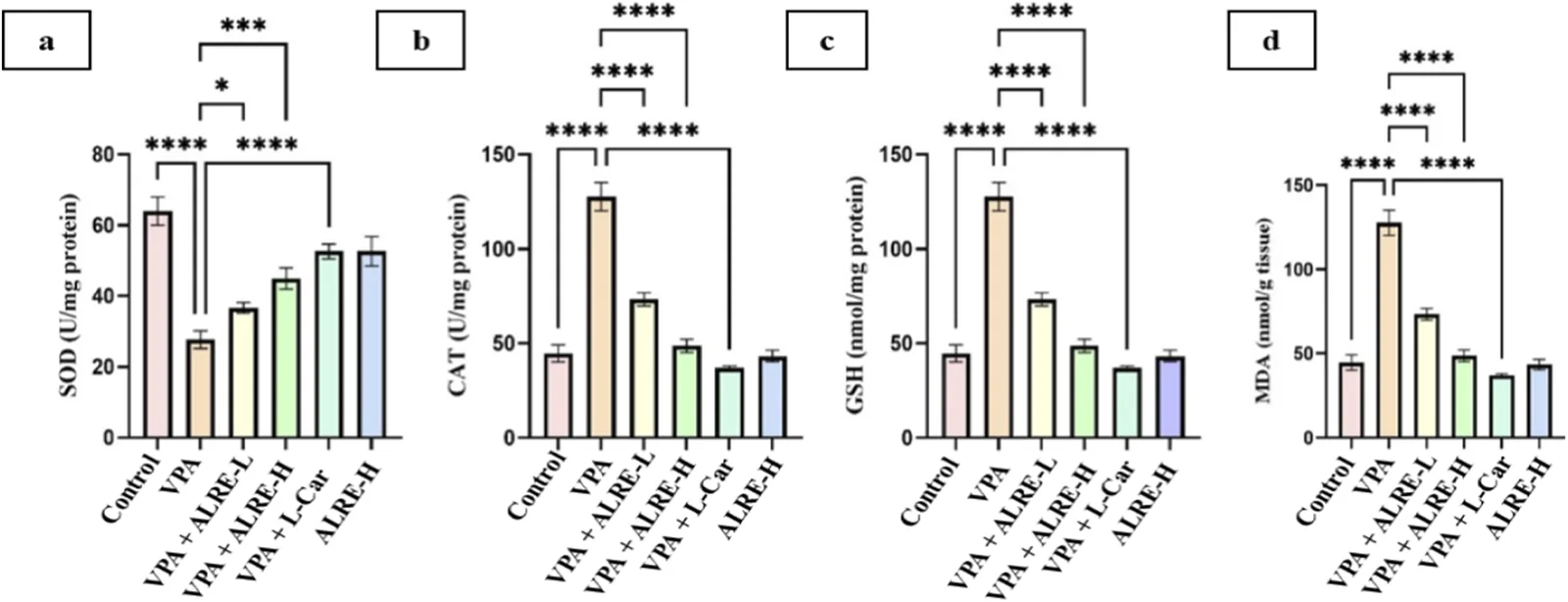
Effects of ALRE and L-carnitine on oxidative stress and antioxidant defence markers in pancreatic tissue of VPA-treated rats. **(a)** Superoxide dismutase (SOD), **(b)** catalase (CAT), **(c)** reduced glutathione (GSH), and **(d)** malondialdehyde (MDA) levels in control, VPA-treated, and treatment groups. Data are expressed as mean ± SEM. Statistical analysis was performed using one-way ANOVA followed by Tukey’s multiple comparisons test. Statistical significance is indicated by asterisks as follows: *p < 0.05, **p < 0.01, ***p < 0.001, ****p < 0.0001. Effect size analysis demonstrated very large treatment effects, with η^2^ values of 0.9015 (CAT), 0.9577 (SOD), 0.9777 (GSH), and 0.9215 (MDA).

### 3.10 Histopathologic evaluation of pancreas

Histopathological analysis showed normal pancreatic structure in Group 1 with healthy tissue, including intact acinar cells, normal ductal epithelium, minimal inflammatory infiltration, and no evidence of fibrosis. Group 2 exhibited moderate pancreatic damage characterised by reduced lobular separation, increased intralobular spaces, mild acinar derangement, and increased early connective tissue proliferation indicative of subacute or unresolved acute pancreatitis. The pancreatic structures of Group 3 were relatively well maintained with intact acinar cells, only slightly reduced islets of Langerhans, and no evidence of necrosis, fibrosis, or inflammation. Group 4 exhibited mild histopathological changes, with only moderately reduced amounts of islet cells with mild cellular derangement. Group 5 demonstrated moderate preservation of pancreatic structure with clearly defined islets and acinar cells with no indication of degeneration, fibrosis, or inflammatory processes. Likewise, Group 6 maintained a normal well-defined healthy pancreatic structure with clearly defined islets and structural intactness of acinar cells with no histopathological changes indicated in Figure 11a. A scoring system was used to assess histopathological alterations to assist in more objectively evaluating changes using a 0–4 scale (0 = normal; 1 = mild; 2 = moderate; 3 = sever; 4 = extensive damage) to evaluate the degree of acinar degeneration, inflammatory infiltration, oedema, necrosis, and architectural abnormality. Three non-overlapping fields were looked at ×10 magnification for each animal, and the mean scores were used for statistical analysis, as shown in Figure 11b.

**FIGURE 11:**
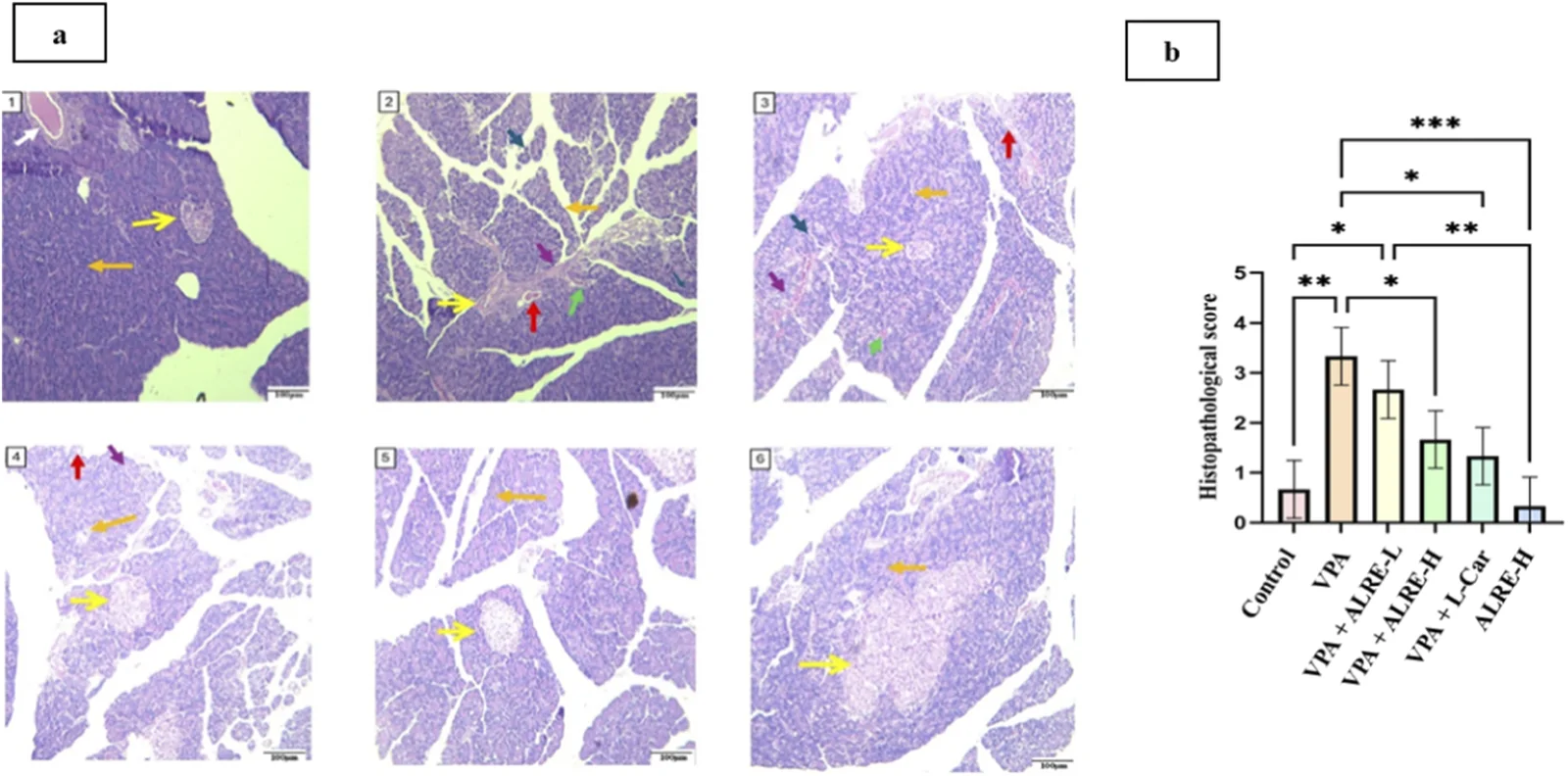
Histopathological evaluation of the pancreatic tissue, as determined using semi-quantitative methods. **(a)** Representative pancreatic tissue sections from experimental Groups 1–6 stained with H&E, demonstrating structural differences across groups. Islets of Langerhans are indicated by yellow arrows, acinar cells by orange arrows, blood vessels by red arrows, interlobular connective tissue septa by purple arrows, fibrotic regions by green arrows, and normal ductal/connective structures by white arrows (Group 1). The degree of pancreatic injury or protection is reflected in changes in islet morphology, acinar organization, vascular congestion, septal thickness, and fibrosis. **(b)** Histopathological scoring that is semi-quantitative (0–4 scale). Mean ± SEM is used to express the data (n = 6). Tukey’s multiple comparison test was used after a one-way ANOVA for statistical analysis (*p < 0.05; **p < 0.01; ***p < 0.001). All images were acquired using identical microscope settings and staining procedures to minimize technical variations and allow direct comparison among groups.

### 3.11 Histopathologic evaluation of liver

The histopathological analysis revealed that Group 1 exhibited normal hepatic architecture, characterized by well-organized radical cords of hepatocytes, intact hepatic sinusoids, and the absence of necrosis, inflammatory cell infiltration, or fibrosis. In Group 2, the tissue was disorganized into lobules; there was swelling of cells caused by fluid accumulation and there was evidence of inflammatory infiltration. In Group 3, evidence of vascular congestion in the central vein and loss of structure of lobules was observed; there was extensive swelling of hepatocytes and damage to the sinusoidal structures and mild-to-moderate inflammatory infiltration. Group 4 contained intact lobular structure; however, this group contained mild amounts of vascular congestion, and only a few vacuolated hepatocytes were noted in the liver tissue. In contrast, Group 5 showed a very similar appearance to Group 6, but there was a slight amount of damage to the liver tissue due to the presence of swelling and open sinusoids space, and Group 5 appeared to be recovering from inflammatory and necrotic processes. The histologically preserved liver structure exhibited by the animals in Group 6 demonstrated that restoration of an intact hepatocellular arrangement and separation of the sinusoidal spaces occurred; thus, the liver from Group 6 appears to be a healthy liver as shown in 12a. A 0-to-4 semi-quantitative scoring scale was established for each animal to allow for more objective scoring of the histological changes. The scoring system was 0, normal; 1, very mild; 2, mild-to-moderate; 3, moderate-to-severe; 4, extensive damage. Evaluation of the various sections for the above-noted factors was conducted for each group. Three non-overlapping fields were looked at for each animal at ×10 magnification, and the mean score was used for statistical analysis, as shown in Figure 12b.

**FIGURE 12:**
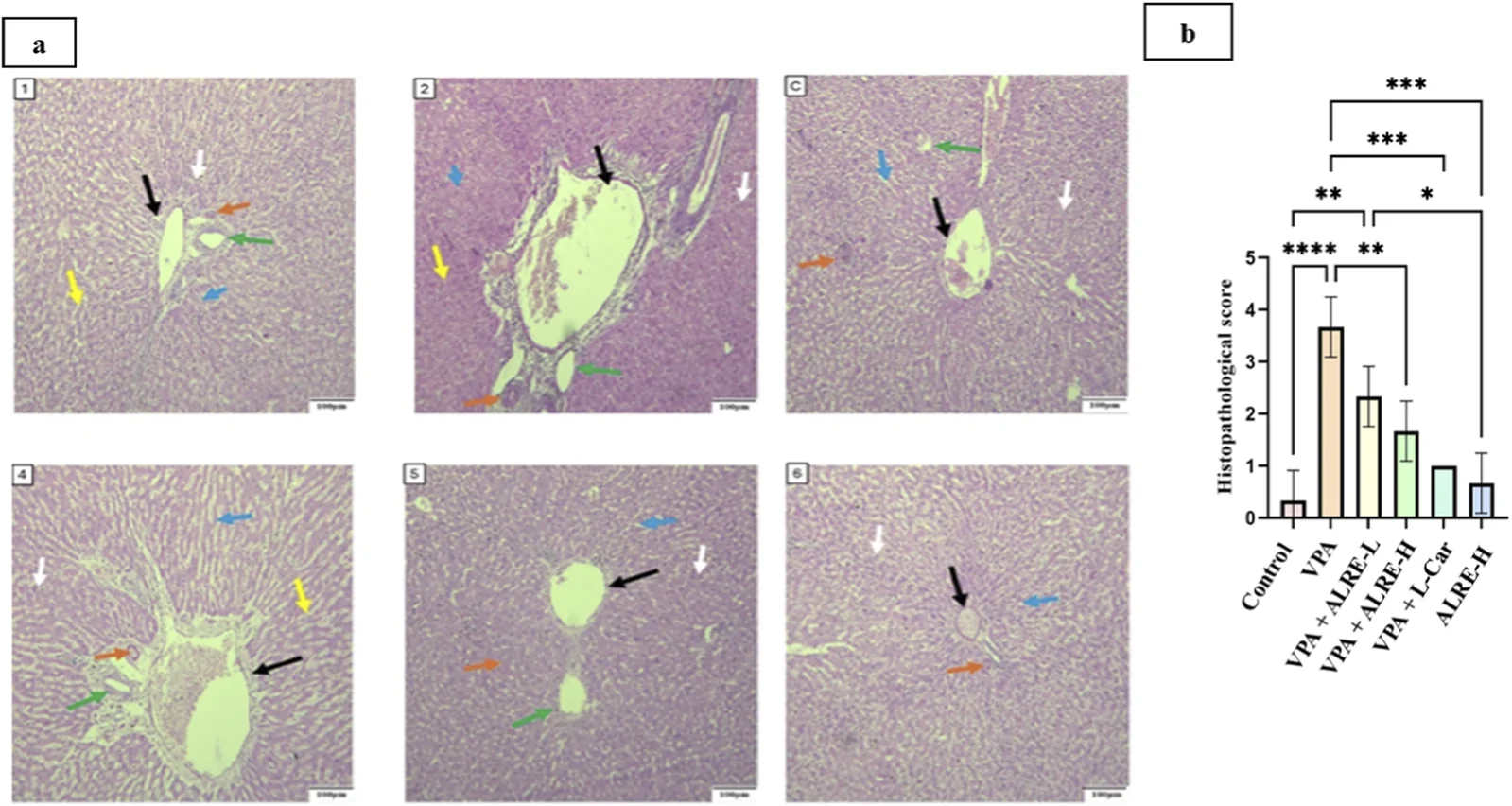
Semi-quantitative histopathological evaluation of liver tissue. **(a)** Liver slices from experimental Groups 1–6 that have been representatively stained with H&E. Central veins are indicated by black arrows, hepatic lobules by green arrows, hepatic sinusoids by blue arrows, bile ducts (portal triad) by orange arrows, binucleated hepatocytes by yellow arrows, and normal hepatocytes by white arrows. The degree of hepatic injury or protection is reflected in structural changes in lobular architecture, sinusoidal organization, and hepatocyte morphology. **(b)** Histopathological scoring that is semi-quantitative (0–4 scale). Mean ± SEM is used to express the data (n = 6). One-way ANOVA and Tukey’s *post hoc* test were used for statistical analysis (*p < 0.05; **p < 0.01; ***p < 0.001). All images were acquired using identical microscope settings and staining procedures to minimize technical variations and allow direct comparison among groups.

### 3.12 Immunohistochemistry analysis of pancreas

Pancreatic tissue analysis using immunohistochemistry for IL-1β showed no detectable IL-1β expression (no brown DAB staining) in groups 1, 5, and 6, thus indicating low inflammatory activity within these groups. In group 2, the presence of IL-1β was seen to be localized to the periductal region and around pancreatic ducts, suggesting the presence of localized inflammation. Mild IL-1β staining was noted in group 3; however, only in the periductal and perivascular areas, and both the islet and acinar areas were found to be negative for IL-1β. Within the periphery of group 4, light to moderate IL-1β staining was found primarily within the connective tissue surrounding the islet and interlobular regions of inflammation. The staining on the endocrine and exocrine portions of the pancreas showed minimal activity with this red antibody. The data above indicate the varying degrees of possible localized inflammatory responses among the experimental groups as presented in Figure 13a. Group 3 (VPA + ALRE 300 mg/kg) exhibited a moderate reduction in pancreatic IL-1β immunoreactivity compared with the VPA-treated group (Group 2). However, the reduction was less pronounced than that observed in Group 4 (VPA + ALRE 500 mg/kg), indicating a dose-dependent anti-inflammatory effect of ALRE.

**FIGURE 13:**
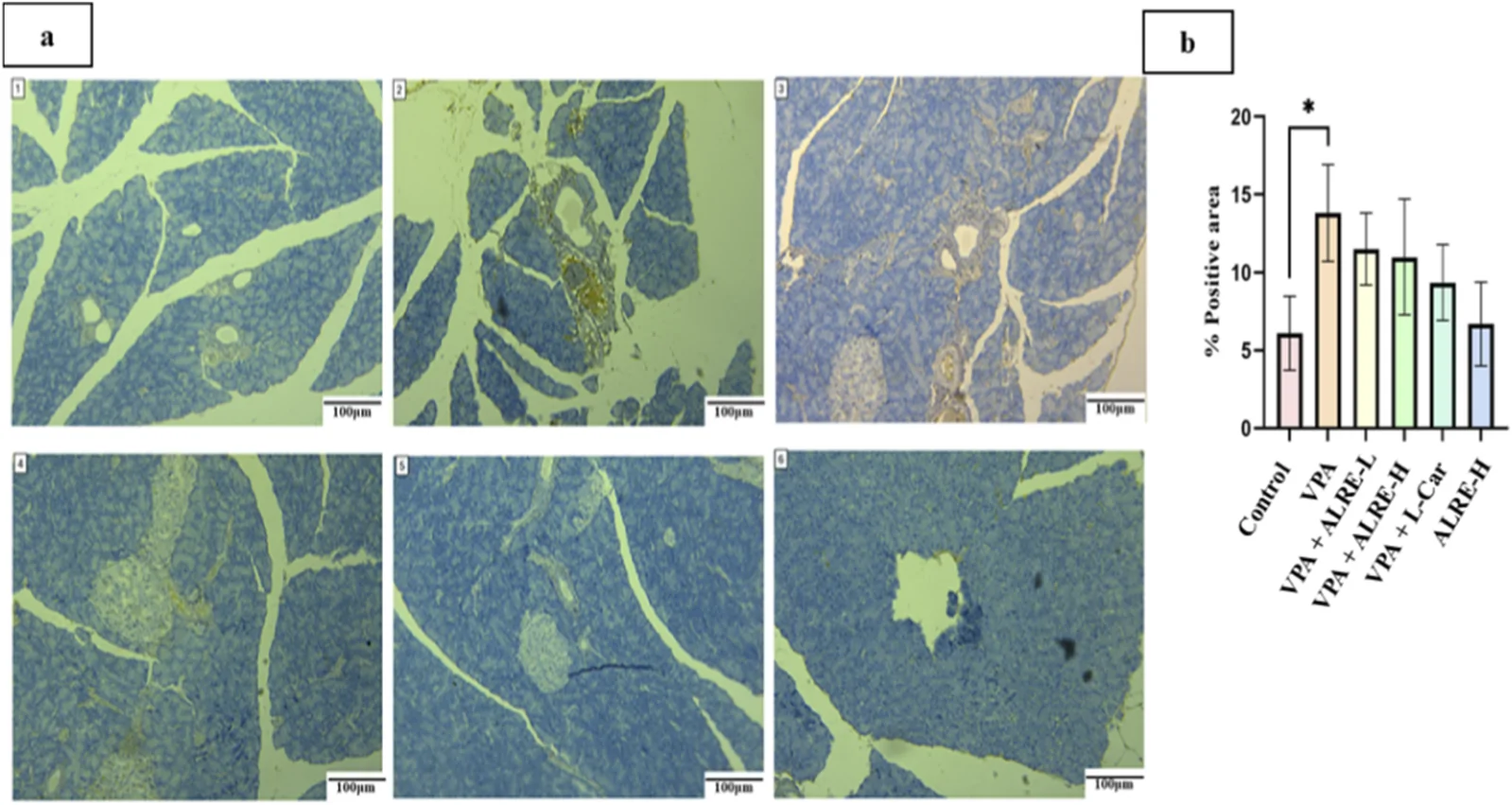
IL-1β immunohistochemical analysis in pancreas tissue. **(a)** Immunohistochemical images showing IL-1β expression from pancreatic sections from various experimental groups: Control, VPA, VPA + ALRE-L, VPA + ALRE-H, VPA + L-Car, and ALRE-H using brown DAB staining for IL-1β positive immunoreactivity with hematoxylin counterstained. Scale bar is 100 µm. **(b)** Quantitative ImageJ analysis of IL-1β immunoreactivity from DAB-positive area as % with means ± SEM (n = 6). Statistical analysis performed using one-way ANOVA with Tukey’s multiple comparison test, *p < 0.05, **p < 0.01, ***p < 0.001.

Immunohistochemical analysis of TNF-α expression in pancreatic tissues showed no detectable immunoreactivity in Groups 1 and 5, with both exocrine and endocrine regions appearing negative. Group 2 exhibited strong TNF-α expression localized around ductal and periductal areas, indicating focal inflammation. Group 3 showed moderate periductal TNF-α staining, with intact islets and acinar tissue showing minimal expression. Group 4 revealed mild TNF-α immunoreactivity confined to ductal and connective tissue regions, whereas Group 6 showed no detectable TNF- α immunoreactivity, with both exocrine and endocrine regions appearing comparable to the control group shown in Figure 14.

**FIGURE 14:**
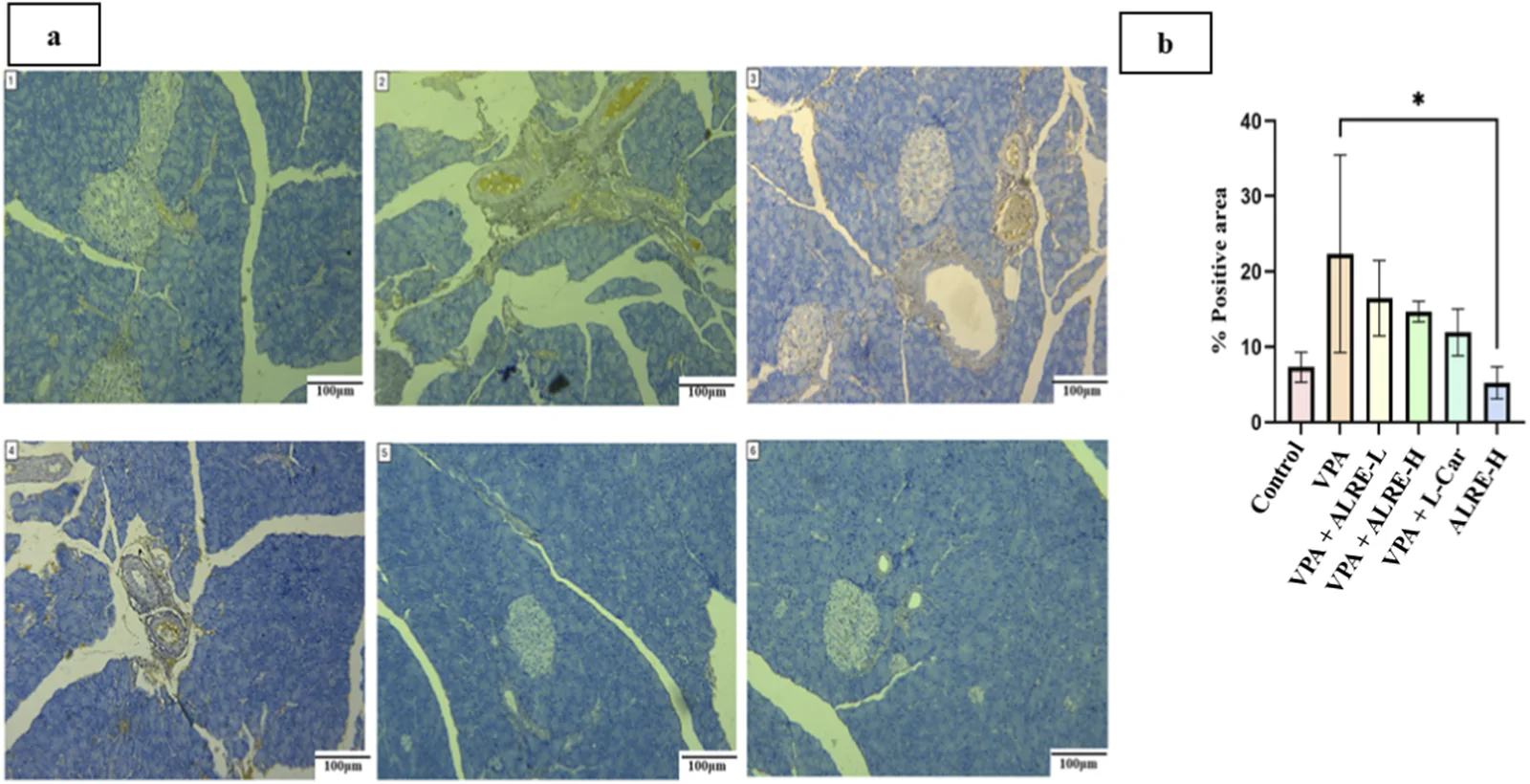
Immunohistochemical examination of TNF-α in pancreas tissue. **(a)** Immunohistochemical images showing TNF-α expression from pancreatic sections from various experimental groups: Control, VPA, VPA + ALRE-L, VPA + ALRE-H, VPA + L-Car, and ALRE-H using brown DAB staining for TNF-α positive immunoreactivity with hematoxylin counterstained. Scale bar is 100 µm. **(b)** Quantitative ImageJ analysis of TNF-α immunoreactivity from DAB-positive area as % with means ± SEM. We used one-way ANOVA and then Tukey’s multiple comparison test to do the statistical analysis. *p < 0.05, **p < 0.01, ***p < 0.001.

### 3.13 Immunohistochemistry analysis of liver

Well-preserved lobular architecture was exhibited in Group 1 liver samples with almost no expression of IL-1β in the Kupffer cells or hepatocytes (the liver cells). The liver samples in Group 2 also displayed some localized expression of IL-1β in the portal indicate some inflammatory activity that has probably come from Kupffer (the cells formed under the liver structures, which differ from hepatocytes) or infiltrating immune cells. Samples of Group 4 showed a low number of IL-1β granules (the presence of IL-1β) primarily found around the central veins and the sinusoids of the livers with evidence of localized Kupffer cell activity, but no active involvement from hepatocytes. Samples in Group 5 exhibited slight to moderate amounts of IL-1β within the peri-central region, which indicates there is a general increase in the activity of Kupffer cells or sinusoid cells. No necrotic tissue was seen. Group 6 exhibited minimal to no IL-1β activity across all zones of the liver, which confirms the lack of inflammatory activation (Figure 15).

**FIGURE 15:**
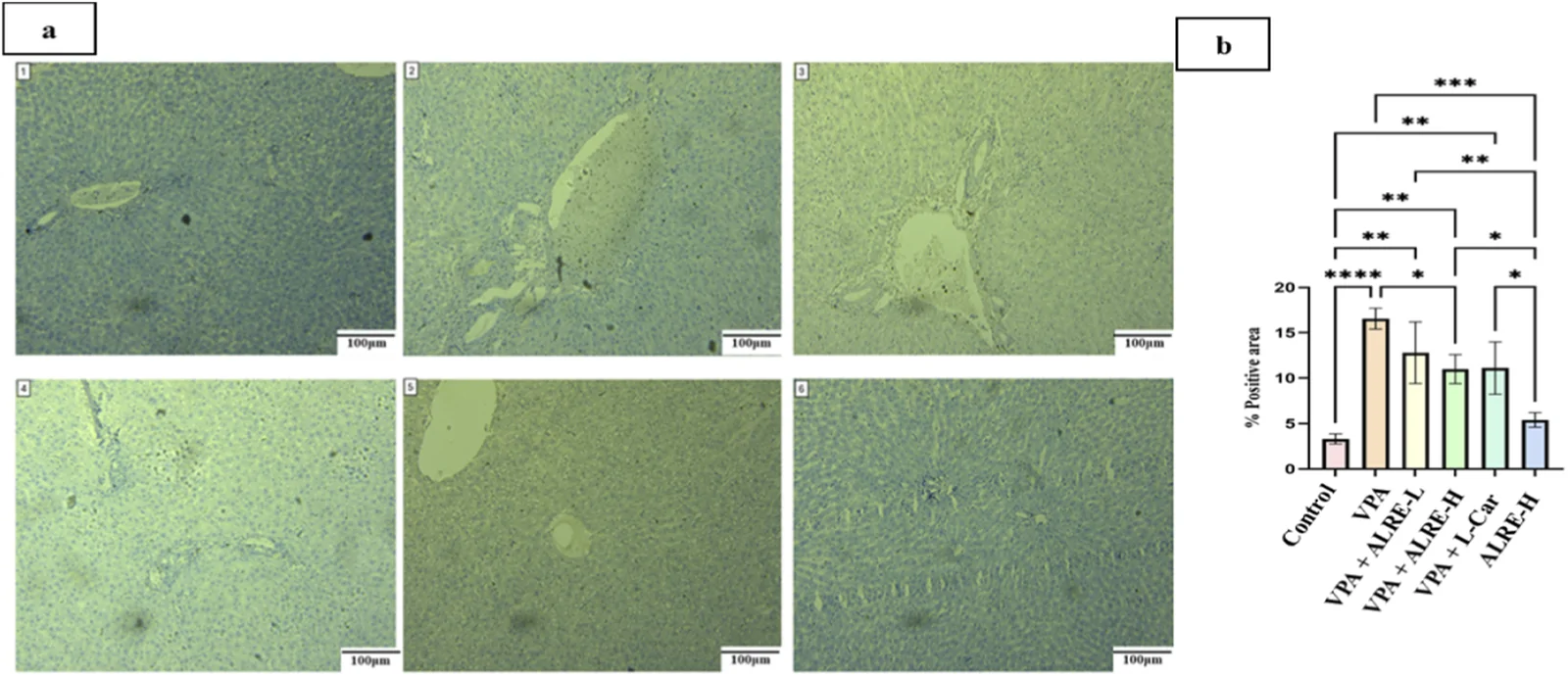
IL-1β immunohistochemical analysis in liver tissue. **(a)** Shows representative images of the IL-1β expression in liver sections derived from different treatment groups Control, VPA, VPA + ALRE-L, VPA + ALRE-H, VPA + L-Car, and ALRE-H. In these figures, the brown stain (DAB) indicates a presence of IL-1β reactivity; this is evident on both the photomicrographs and histological slide of the liver. All photomicrographs are x100. In the top section of the photomicrographs, Cell Nuclei are stained with Hematoxylin. The scale bar represents 100 µm. **(b)** Quantitative analysis of IL-1β immunoreactivity using ImageJ. Data are expressed as a mean ± SEM (n = 6). One-way ANOVA followed by Tukey’s post-hoc test was performed on the quantitative data. Statistically significant differences were determined by asterisks (* <0.05, ** <0.01, *** <0.001).

Hepatic structure was not damaged in Group 1 control, and there was little TNF-α (in only a few scattered granules), consistent with normal Kupffer cell activity. Group 2 had an increase in TNF-α staining indicating that inflammation, and therefore injury, was present. TNF-α staining occurred in the area surrounding the central vein, and the portal vein’s blood vessels, as well as the presence of a fibrinous debris, suggested a localized inflammatory response. TNF-α staining in Group 4 was noted primarily in the periportal region marked by Kupffer cells and sinusoids. The liver parenchyma appeared intact. Therefore, this was attributed to a mild, localized immune response to a stressful event. Only minimal amounts of TNF-α staining were detected in Group 5’s centrilobular area; thus, minimal non-specific TNF-α staining in this group suggested no evidence of inflammation. Finally, Group 6 had no TNF-α staining whatsoever, confirming that there was no evidence of liver inflammation and that liver histology was fully preserved, as depicted in Figure 16.

**FIGURE 16:**
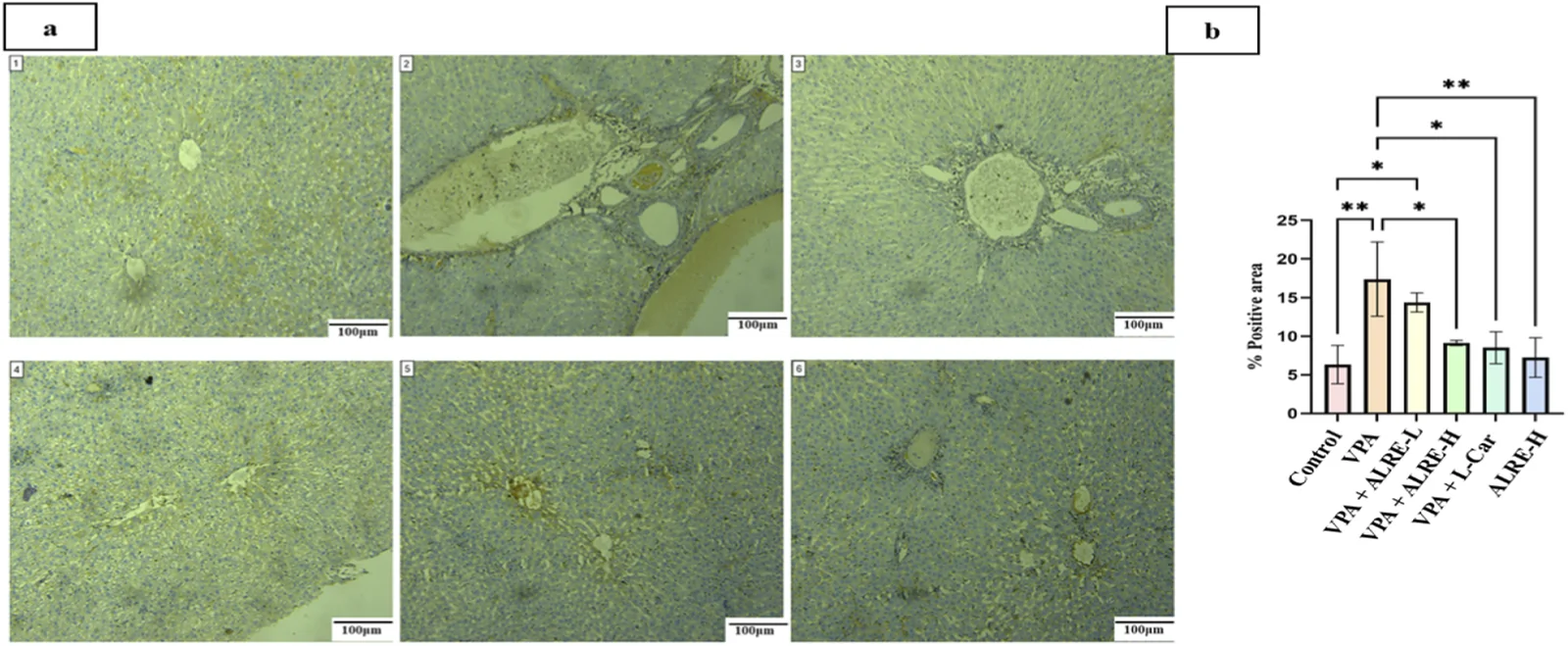
TNF-α immunohistochemical analysis in liver tissue. **(a)** The images presented as TNF-α expression levels in liver sections taken from various experimental groups: Control, VPA, VPA + ALRE-L, VPA + ALRE-H, VPA + L-Car, and ALRE-H. Brown DAB staining indicates TNF-α protein expression in tissues, and the blue (or purple) haematoxylin staining indicates cell nucleus position. Scale bar = 100 µm. **(b)** Graphically shows the quantitative measurement of TNF-α immunoreactivity using ImageJ as a percentage of the DAB positive area (mean ± SEM) (n = 6). Results were analysed using one-way ANOVA with Tukey’s multiple comparison test; *p < 0.05; **p < 0.01; ***p < 0.001.

## 4 Discussion

The capacity of valproic acid (valproate; VPA) to cause damage to the liver and pancreas through both oxidative stress and inflammation has been widely documented. In the present study, significant protection from VPA hepatopancreatic toxicity by ALRE was observed through the measurement of serum enzymes, the restoration of the body’s antioxidant systems, the inhibition of lipid peroxidation, and the decrease in the level of proinflammatory cytokines (TNF-α and IL-1β). The VPA dose (300 mg/kg b.w/day, p.o.) was selected based on the previous studies demonstrating consistent induction of hepatotoxicity and pancreatitis in rodent models. This dose has been widely employed to reproduce biochemical, oxidative and histopathological alterations associated with VPA-induced organ toxicity. Where direct extrapolation of this dose to human is limited by interspecies differences in metabolism and drug clearance. Nevertheless, the biochemical and histopathological changes observed in the present study closely resembles the hepatic and pancreatic abnormalities reported in human VPA toxicity, supporting the translational relevance of this experimental model.

The hepatoprotective and pancreatoprotective effects of ALRE observed in the present study are likely mediated through its antioxidant and anti-inflammatory properties. VPA-induced toxicity is characterized by mitochondrial dysfunction, excessive generation of ROS, lipid peroxidation, and activation of inflammatory pathways, ultimately leading to hepatic and pancreatic injury. The restoration of endogenous antioxidants defences, reduction in MDA levels, and decreased expression of TNF-α and IL-1β observed following ALRE treatment suggest that the extract mitigates oxidative and inflammatory damage and preserve tissue integrity. However, the precise molecular pathways underlying these effects were not directly investigated and warrant further study. The histological protection seen in the ALRE-treated groups provides additional evidence of this protective role. Additionally, our findings are consistent with previous research illustrating the antioxidant and anti-inflammatory activity of *Arctium lappa* in a variety of other models of damage from toxicants. For example, it was shown that amyl nitrite, acetaminophen, cadmium, lead, chromium (VI), and radiation have also caused oxidative stress, histopathological damage, and increases in inflammatory cytokines when exposed to *A. lappa* extracts. However, those studies should not be regarded as directly applicable to the present study, as they were done under different types of experimental conditions than those used here.

To best of our knowledge, this is the first study to demonstrate the protective effects of ALRE against VPA-induced hepatopancreatic toxicity. The findings extend previous reports describing the antioxidant and anti-inflammatory activities of *A. lappa* in other toxicological models and provide evidence supporting its potential therapeutic application in drug-induced organ injury. Injury from VPA has a unique biochemical profile of mitochondrial dysfunction and cytokine-mediated inflammation, our results support hepatoprotectives of ALRE for this clinically relevant VPA toxicity induced by drug therapy.

Another factor to consider is the way ALRE might affect the way VPA is metabolised. VPA is metabolised in the liver by mitochondrial β-oxidation, cytochrome p450–mediated ω-oxidation, and carnitine-dependent processes. Previous studies have suggested that phytochemicals present in *Arctium lappa* may modulate oxidative stress and mitochondrial function (Farzaei et al., 2025). Nevertheless, the present study did not evaluate pharmacokinetics, cytochrome P450 activity, mitochondrial β-oxidation; therefore, the possibility that ALRE influences VPA metabolism remains speculative and require further investigation. However, speculation regarding these interactions is limited within the context of this study as we did not conduct pharmacokinetic measurements, cytochrome activity assays, or evaluate β-oxidation or carnitine levels.

The current work assessed the safety profile of ALRE in normal liver (THLE-2) and pancreatic (H6C7) cell lines, while its cytotoxic effects were assessed in Hep3B and PANC-1 cancer cell lines. Additionally, the extract’s protective effects were confirmed *in vivo* using a model of pancreatitis and hepatotoxicity caused by valproic acid. Although the findings translational value is strengthened by the inclusion of both malignant and normal cell lines and *in vivo* validation. Previous studies using *A. lappa* have suggested the role of antioxidant and anti-inflammatory pathways such as NF-κB, Nrf2, and GSK-3β; our study, however, did not directly examine these pathways (Shyam and Sabina, 2024; Yang et al., 2025). Therefore, any references to these pathways are made with caution and should be considered as speculative. The observed reduction in MDA, TNF-α and IL-1β levels following ALRE treatment is consistent with attenuation of oxidative stress and inflammatory signaling as shown in Figure 17. Previous studies have implicated pathways such as NF-κB, Nrf2, and GSK-3β in the biological activities of *A. lappa*; however, these signaling pathways were not directly examined in the present study. The histopathological findings suggested a dose-dependent protective effect of ALRE against VPA-induced liver injury, with the higher dose (500 mg/kg) exhibiting greater preservation of hepatic architecture than the lower dose (300 mg/kg). The apparent difference between groups 3 and 4 may reflect biological variability associated with small sample size and inter-animal differences. Although the 500 mg/kg dose showed greater protection overall, further studies with large sample sizes are required to confirm the dose-response relationship.

**FIGURE 17:**
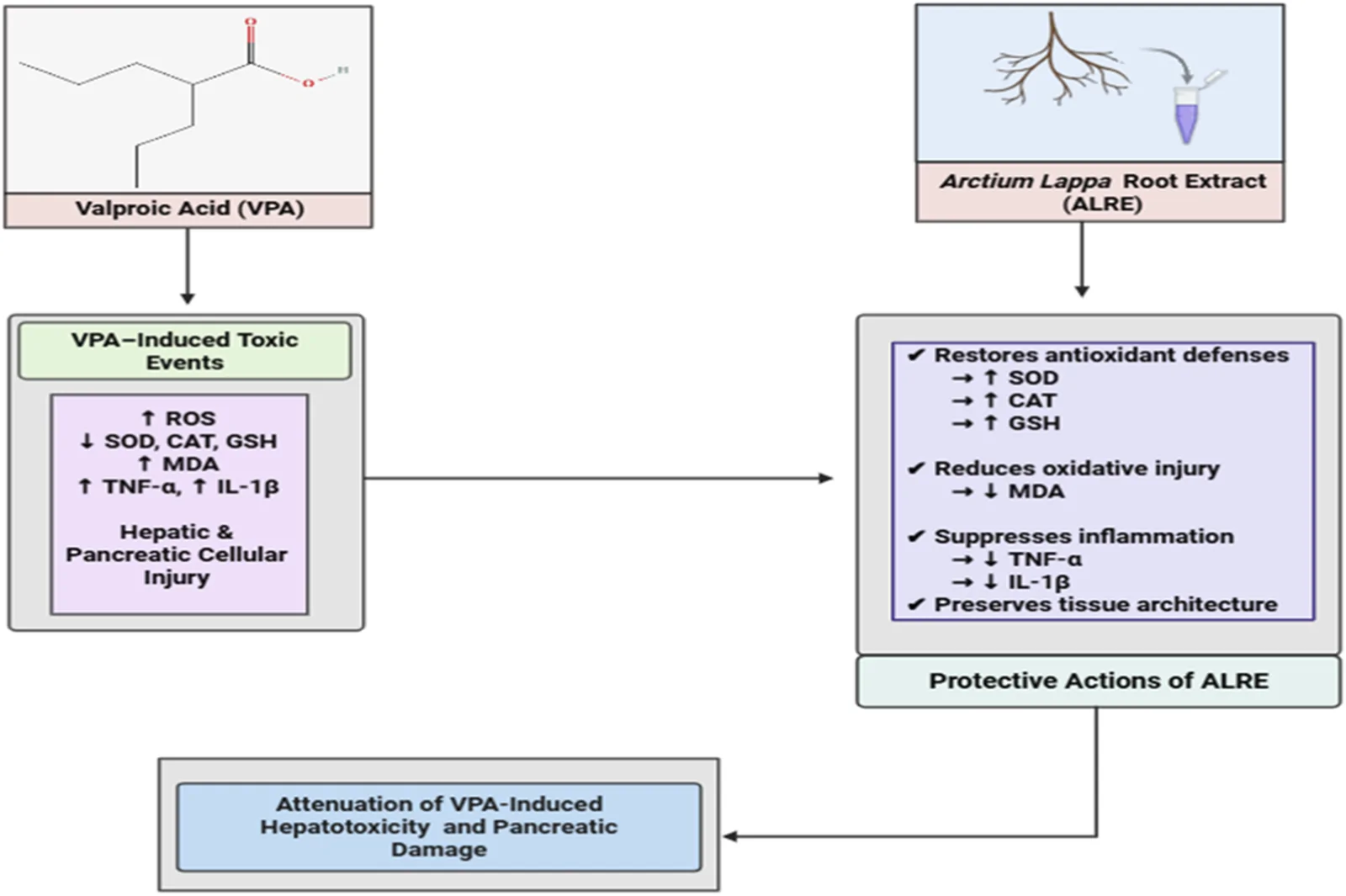
A diagram showing how ALRE protects against VPA-induced hepatopancreatic toxicity by restoring antioxidant defences, lowering oxidative stress and inflammatory indicators, and keeping tissue structure intact.

There are several limitations to the current work. To begin with, the cytotoxicity evaluation was carried out on a single time-point (24 h), meaning that subsequent research must consider different exposure periods (48 and 72 h) to identify the time-dependent pattern of cytotoxicity caused by ALRE. Secondly, even though food and water intake was monitored regularly for the purpose of protecting the experimental animals, these parameters were not quantitatively recorded and, consequently, could not be included in the analysis. Thirdly, the current study concentrated only on acute hepatotoxicity and pancreatitis caused by VPA, which limits the assessment of the long-term protective properties of ALRE. Furthermore, the pharmacokinetics and possible herb-drug interactions due to ALRE have not been investigated. Fourthly, the mechanistic investigation has been limited to biochemical and immunohistochemical indicators of inflammation and oxidative stress but not molecular validation. Even though the number of animals in each group did not exceed six due to ethical and logistical restrictions, the observed differences among different experimental groups were statistically significant. Future studies employing larger sample sizes together with molecular and transcriptomic approaches are warranted to validate these findings and further elucidate the underlying signaling pathways and therapeutic potential of ALRE in clinical settings.

Future studies will focus on elucidating the molecular mechanisms underlying the protective effects of ALRE using western blotting and qPCR analyses of key pathways involved in oxidative stress, inflammation, and apoptosis. Furthermore, pharmacokinetic interactions between ALRE and VPA, including the quantification of toxic metabolites such as 4-ene-VPA and 2,4-diene-VPA using LC-MS/MS techniques will be investigated. Such studies will help determine whether the protective effects of ALRE are mediated solely by antioxidant and anti-inflammatory mechanisms or also involve modulation of VPA metabolism and mitochondrial β-oxidation.

## 5 Conclusion

ALRE appears to mitigate the hepatopancreatic damage caused by VPA in Wistar rats; that is achieved through its use of the oxidative stress and inflammatory markers associated with VPA, as well as through the preservation of hepatic and pancreatic histology. Treatment with ALRE significantly reduced the elevated serum levels of hepatic and pancreatic enzymes induced by VPA. In addition, ALRE restored antioxidant defence systems, including SOD, CAT, and GSH, reduced lipid peroxidation as indicated by decreased MDA levels, and attenuated the expression of the pro-inflammatory cytokines TNF-α and IL-1β. The dose of ALRE that had the most potent antioxidant and anti-inflammatory effects (i.e., protective effects) was the 500 mg/kg, which is like that of L-carnitine; this reinforces the previous findings and demonstrates that ALRE has a significant protective effect in a particular model of VPA-induced toxicity. Considering the literature available pertaining to the antioxidant and anti-inflammatory properties of *A. lappa* extract, inputting the present results with the reported evidence from previous studies that have provided similar results is useful in developing the understanding of how the protective properties of ALRE work in conjunction with the current study results. However, the previous research examined the effects of ALRE on the hepatic and pancreatic tissue damage induced by a different toxic agent and using a different experimental model than the present research on the protective effects of ALRE in a VPA-induced experimental model of hepatotoxicity and pancreatitis. Therefore, the contribution of the current study is the further validation of the protective effects of ALRE in a model of hepatopancreatic damage induced by VPA; as such, it provides additional support for-the-targets of-ALRE-based therapeutics. In addition to these findings, the present study also has several limitations.

The study described herein lacks an analysis of the detailed molecular processes, pharmacokinetic analysis, assessment of chronic toxicity, and interaction between herbal medicines and prescription drugs; all of which must be examined before considering possible application into clinical medicine. The findings of the experiment are limited strictly to the acute response of rodents to the dosing conditions used during this study and should not be generalized to humans without substantial caution. Future studies should include a pathway-based analysis of the results, investigation of dose-response relationships, establishment of safety profiles, assessment of long-term exposure to dose-response relationships, and evaluation of possible interactions with currently used medications for the treatment of epilepsy. These types of studies are crucial for understanding the mechanistic basis, therapeutic window, and clinical relevance of ALRE.

## Data Availability

The raw data supporting the conclusions of this article will be made available by the authors, without undue reservation.
