# Supplementary material for: Hepatoprotective and pancreatoprotective effects of Arctium lappa root extract in valproic acid–induced toxicity: biochemical, histopathological, and immunohistochemical analysis
Source: Front Pharmacol. 2026 Oct 16;17:1883451. doi: 10.3389/fphar.2026.1883451 (PMC13719420; doi:10.3389/fphar.2026.1883451)
Supplement: Supplementary file 1 [file DataSheet1.pdf]

NOTE: Please save this file locally before filling in the table, DO NOT work on the file within your internet browser as changes will not be saved. Adobe Acrobat Reader (available free here) is recommended for completion.

The ARRIVE guidelines 2.0: author checklist

The ARRIVE Essential 10

These items are the basic minimum to include in a manuscript. Without this information, readers and reviewers cannot assess the reliability of the findings.

Item		Recommendation	Section/line number, or reason for not reporting
**Study design**	1	For each experiment, provide brief details of study design including:<br>a. The groups being compared, including control groups. If no control group has been used, the rationale should be stated.<br>b. The experimental unit (e.g. a single animal, litter, or cage of animals).	Thirty-six male Wistar albino rats were randomly divided into six groups (n = 6 per group), including normal control, disease control, standard control, and treatment groups.<br>Each individual rat was considered as the experimental unit.
**Sample size**	2	a. Specify the exact number of experimental units allocated to each group, and the total number in each experiment. Also indicate the total number of animals used.<br>b. Explain how the sample size was decided. Provide details of any *a priori* sample size calculation, if done.	Six animals were allocated per group (n = 6), with a total of 36 animals used in the experiment.<br>Sample size was determined based on previously published similar experimental studies. No formal a priori power calculation was performed.
**Inclusion and exclusion criteria**	3	a. Describe any criteria used for including and excluding animals (or experimental units) during the experiment, and data points during the analysis. Specify if these criteria were established *a priori*. If no criteria were set, state this explicitly.<br>b. For each experimental group, report any animals, experimental units or data points not included in the analysis and explain why. If there were no exclusions, state so.<br>c. For each analysis, report the exact value of *n* in each experimental group.	No predefined inclusion or exclusion criteria were established prior to the study.<br>All 36 animals completed the experimental protocol and were included in the final analysis. No animals or data points were excluded.<br>For all analyses, the exact value of n was 6 per group.
**Randomisation**	4	a. State whether randomisation was used to allocate experimental units to control and treatment groups. If done, provide the method used to generate the randomisation sequence.<br>b. Describe the strategy used to minimise potential confounders such as the order of treatments and measurements, or animal/cage location. If confounders were not controlled, state this explicitly.	Animals were randomly allocated into six experimental groups (n = 6 per group) using a simple randomisation method to ensure unbiased distribution.<br>Cage placement and order of handling were rotated periodically to minimise environmental and handling-related confounding factors.
**Blinding**	5	Describe who was aware of the group allocation at the different stages of the experiment (during the allocation, the conduct of the experiment, the outcome assessment, and the data analysis).	Investigators performing biochemical assays and histopathological evaluations were blinded to group allocation during outcome assessment and data analysis.
**Outcome measures**	6	a. Clearly define all outcome measures assessed (e.g. cell death, molecular markers, or behavioural changes).<br>b. For hypothesis-testing studies, specify the primary outcome measure, i.e. the outcome measure that was used to determine the sample size.	Primary outcome measures included serum biochemical markers (ALT, AST, amylase), oxidative stress parameters (SOD, CAT, GSH, MDA), apoptotic markers (Caspase-3, Bcl-2), and histopathological scoring.<br>The primary outcome measures were used to assess hepatoprotective and pancreatoprotective effects.
**Statistical methods**	7	a. Provide details of the statistical methods used for each analysis, including software used.<br>b. Describe any methods used to assess whether the data met the assumptions of the statistical approach, and what was done if the assumptions were not met.	Data were exp<br>Normality of data distribution was assessed prior to analysis. A p-value < 0.05 was considered statistically significant.
**Experimental animals**	8	a. Provide species-appropriate details of the animals used, including species, strain and substrain, sex, age or developmental stage, and, if relevant, weight.<br>b. Provide further relevant information on the provenance of animals, health/immune status, genetic modification status, genotype, and any previous procedures.	Healthy adult male Wistar albino rats (Rattus norvegicus) were used in the study. The animals were 8–10 weeks old, representing young adult rats, and weighed approximately 180–220 g at the beginning of the experiment. Only male rats were included to<br>Animals were procured from a CPCSEA-registered animal facility and had no prior experimental exposure.
**Experimental procedures**	9	For each experimental group, including controls, describe the procedures in enough detail to allow others to replicate them, including:<br>a. What was done, how it was done and what was used.<br>b. When and how often.<br>c. Where (including detail of any acclimatisation periods).<br>d. Why (provide rationale for procedures).	After acclimatisation, treatments were administered orally according to group allocation for 28 days.<br>Drug doses, route of administration (oral gavage), frequency, and disease induction protocols are described in Sections 2.4–2.7.<br>On the final da<br>The 28-day oral treatment protocol using VPA (300 mg/kg) was designed to induce sustained hepatotoxicity and pancreatic injury via oxidative and mitochondrial mechanisms, while ALRE and L-carnitine were
**Results**	10	For each experiment conducted, including independent replications, report:<br>a. Summary/descriptive statistics for each experimental group, with a measure of variability where applicable (e.g. mean and SD, or median and range).<br>b. If applicable, the effect size with a confidence interval.	Data are prese<br>Statistical significance and comparative analyses between groups are reported in the Results section.

The Recommended Set

These items complement the Essential 10 and add important context to the study. Reporting the items in both sets represents best practice.

Item		Recommendation	Section/line number, or reason for not reporting
**Abstract**	11	Provide an accurate summary of the research objectives, animal species, strain and sex, key methods, principal findings, and study conclusions.	This study evaluated the protective effects of A
**Background**	12	a. Include sufficient scientific background to understand the rationale and context for the study, and explain the experimental approach.<br>b. Explain how the animal species and model used address the scientific objectives and, where appropriate, the relevance to human biology.	Valproic acid (VPA) is associated with hepatotoxicity and pancreatitis due to oxidative stress and mitochondrial dysfunction. Arctium lappa contains bioactive compounds with antioxidant and anti-inflammatory properties, providing a<br>Male Wistar rats were selected due to their well-established use in toxicological studies and predictable response to hepatotoxic agents. The VPA-induced model mimics key mechanisms involved in human drug-induced liver and pancreatic injury, offering
**Objectives**	13	Clearly describe the research question, research objectives and, where appropriate, specific hypotheses being tested.	Research Question: Does Arctium lappa root e
**Ethical statement**	14	Provide the name of the ethical review committee or equivalent that has approved the use of animals in this study, and any relevant licence or protocol numbers (if applicable). If ethical approval was not sought or granted, provide a justification.	All experimental procedures were reviewed and approved by the Institutional Animal Ethics Committee (IAEC) of Vellore Institute of Technology (VIT), constituted under CPCSEA guidelines, Government of India, under Certificate No: VIT/IAEC/17Apr/24/26. All animal experiments were conducted in accordance with CPCSEA regulations and institutional ethical standards for laboratory animal care and use.
**Housing and husbandry**	15	Provide details of housing and husbandry conditions, including any environmental enrichment.	Male Wistar albino rats were housed in polypro
**Animal care and monitoring**	16	a. Describe any interventions or steps taken in the experimental protocols to reduce pain, suffering and distress.<br>b. Report any expected or unexpected adverse events.<br>c. Describe the humane endpoints established for the study, the signs that were monitored and the frequency of monitoring. If the study did not have humane endpoints, state this.	Animals were monitored daily for signs of distress or toxicity.<br>No unexpected adverse events were observed during the study.<br>No animals required early euthanasia.
**Interpretation/ scientific implications**	17	a. Interpret the results, taking into account the study objectives and hypotheses, current theory and other relevant studies in the literature.<br>b. Comment on the study limitations including potential sources of bias, limitations of the animal model, and imprecision associated with the results.	The results support the hypothesis that VPA induces hepatic and pancreatic injury through oxidative stress and mitochondrial dysfunction, while ALRE significantly mitigated these effects, likely via antioxidant and cytoprotective mechanisms. The findings are<br>The study is limited by the use of a single animal species and sex, a relatively small sample size, and reliance mainly on biochemical and histological markers without detailed molecular validation. Additionally, species differences in drug metabolism may
**Generalisability/ translation**	18	Comment on whether, and how, the findings of this study are likely to generalise to other species or experimental conditions, including any relevance to human biology (where appropriate).	Because oxida
**Protocol registration**	19	Provide a statement indicating whether a protocol (including the research question, key design features, and analysis plan) was prepared before the study, and if and where this protocol was registered.	No protocol registration was performed prior to the study.
**Data access**	20	Provide a statement describing if and where study data are available.	Study data are available from the correspondir
**Declaration of interests**	21	a. Declare any potential conflicts of interest, including financial and non-financial. If none exist, this should be stated.<br>b. List all funding sources (including grant identifier) and the role of the funder(s) in the design, analysis and reporting of the study.	The authors declare no conflicts of interest.<br>No Funding
