# Supplementary material for: Hepatoprotective and pancreatoprotective effects of Arctium lappa root extract in valproic acid–induced toxicity: biochemical, histopathological, and immunohistochemical analysis
Source: Front Pharmacol. 2026 Oct 16;17:1883451. doi: 10.3389/fphar.2026.1883451 (PMC13719420; doi:10.3389/fphar.2026.1883451)
Supplement: Supplementary file 2 [file Supplementaryfile1.docx]

**Hepatoprotective and Pancreatoprotective Effects of *Arctium lappa* Root Extract in Valproic Acid–Induced Toxicity: Biochemical, Histopathological, and Immunohistochemical Analysis**

Mukul Shyam and Sabina Evan Prince*

*Department of Biotechnology, School of Biosciences and Technology, VIT University,*

*Vellore, Tamil Nadu, India*

Valproic acid Reported Potential Side Effects

(Vigiaccess-WHO)

**GC-MS Chromatogram**

**HPLC Chromatogram**

**COHEN’s Values**

**For ALT**

| **COMPARISONS (with Control)** | **COHEN’S D VALUE** |
| --- | --- |
| VPA | 13.399 |
| VPA + ALRE-L | 7.088 |
| VPA + ALRE-H | 0.988 |
| VPA + L-CAR | 2.346 |
| ALRE-H | 0.346 |

**For AST**

| **COMPARISONS (with Control)** | **COHEN’S D VALUE** |
| --- | --- |
| VPA | 9.849 |
| VPA + ALRE-L | 5.564 |
| VPA + ALRE-H | 2.179 |
| VPA + L-CAR | 0.241 |
| ALRE-H | 2.362 |

**For ALP**

| **COMPARISONS (with Control)** | **COHEN’S D VALUE** |
| --- | --- |
| VPA | 9.849 |
| VPA + ALRE-L | 5.564 |
| VPA + ALRE-H | 2.179 |
| VPA + L-CAR | 0.241 |
| ALRE-H | 2.362 |

**For GGT**

| **COMPARISONS (with Control)** | **COHEN’S D VALUE** |
| --- | --- |
| VPA | 23.403 |
| VPA + ALRE-L | 10.322 |
| VPA + ALRE-H | 6.513 |
| VPA + L-CAR | 2.996 |
| ALRE-H | 5.712 |

**For Serum Amylase**

| **COMPARISONS (with Control)** | **COHEN’S D VALUE** |
| --- | --- |
| VPA | 8.217 |
| VPA + ALRE-L | 7.518 |
| VPA + ALRE-H | 3.946 |
| VPA + L-CAR | 4.206 |
| ALRE-H | 1.811 |

**For Serum Lipase**

| **COMPARISONS (with Control)** | **COHEN’S D VALUE** |
| --- | --- |
| VPA | 6.305 |
| VPA + ALRE-L | 8.060 |
| VPA + ALRE-H | 5.422 |
| VPA + L-CAR | 2.375 |
| ALRE-H | 1.853 |

**For Liver SOD**

| **COMPARISONS (with Control)** | **COHEN’S D VALUE** |
| --- | --- |
| VPA | 11.049 |
| VPA + ALRE-L | 8.397 |
| VPA + ALRE-H | 4.553 |
| VPA + L-CAR | 3.168 |
| ALRE-H | 2.935 |

**For Liver CAT**

| **COMPARISONS (with Control)** | **COHEN’S D VALUE** |
| --- | --- |
| VPA | 10.975 |
| VPA + ALRE-L | 6.738 |
| VPA + ALRE-H | 4.058 |
| VPA + L-CAR | 1.767 |
| ALRE-H | 3.136 |

**For Liver GSH**

| **COMPARISONS (with Control)** | **COHEN’S D VALUE** |
| --- | --- |
| VPA | 6.926 |
| VPA + ALRE-L | 3.964 |
| VPA + ALRE-H | 2.652 |
| VPA + L-CAR | 1.413 |
| ALRE-H | 1.625 |

**For Liver MDA**

| **COMPARISONS (with Control)** | **COHEN’S D VALUE** |
| --- | --- |
| VPA | 14.353 |
| VPA + ALRE-L | 6.798 |
| VPA + ALRE-H | 4.520 |
| VPA + L-CAR | 4.256 |
| ALRE-H | 3.160 |

**For Pancreas SOD**

| **COMPARISONS (with Control)** | **COHEN’S D VALUE** |
| --- | --- |
| VPA | 10.87 |
| VPA + ALRE-L | 9.02 |
| VPA + ALRE-H | 5.37 |
| VPA + L-CAR | 3.55 |
| ALRE-H | 2.77 |

**For Pancreas CAT**

| **COMPARISONS (with Control)** | **COHEN’S D VALUE** |
| --- | --- |
| VPA | 9.65 |
| VPA + ALRE-L | 4.77 |
| VPA + ALRE-H | 1.24 |
| VPA + L-CAR | 1.82 |
| ALRE-H | 1.07 |

**For Pancreas GSH**

| **COMPARISONS (with Control)** | **COHEN’S D VALUE** |
| --- | --- |
| VPA | 18.72 |
| VPA + ALRE-L | 6.83 |
| VPA + ALRE-H | 9.2 |
| VPA + L-CAR | 5.48 |
| ALRE-H | 1.4 |

**For Pancreas MDA**

| **COMPARISONS (with Control)** | **COHEN’S D VALUE** |
| --- | --- |
| VPA | 5.65 |
| VPA + ALRE-L | 4.8 |
| VPA + ALRE-H | 2.65 |
| VPA + L-CAR | 1.74 |
| ALRE-H | 0.19 |
